# Deciphering the nitrogen-promoting effect of vermicompost: from a complex organic matrix to synthetic microbial communities

**DOI:** 10.3389/fpls.2026.1946342

**Published:** 2026-09-28

**Authors:** Yangfei Li, Jing Huang, Yuanke Liang

**Affiliations:** 1 College of Life Sciences and Agri-forestry, Southwest University of Science and Technology, Mianyang, China; 2 i-Microbiome Lab (Intelligent Microbiome and Bio-Manufacturing Laboratory), Southwest University of Science and Technology, Mianyang, China

**Keywords:** microbial community, nitrogen-use efficiency, organic fertilizer, synthetic microbial community (SynCom), vermicompost

## Abstract

Excessive application of chemical nitrogen fertilizers and low utilization efficiency are key issues constraining sustainable agricultural development, and the development of green nitrogen-regulating inputs is a core requirement for the green transformation of agriculture. Vermicompost, produced through the synergistic conversion of organic waste by earthworms and microorganisms, possesses both excellent nitrogen retention capacity and an active microbial community, demonstrating substantial capacity for improving soil nitrogen-use efficiency. Existing research indicates that the combined application of vermicompost and chemical fertilizers can significantly improve crop nitrogen-use efficiency and yield performance. However, there remains a lack of systematic analysis regarding the independent contributions of their matrix and microbial components, as well as the mechanisms underlying their synergistic effects; the pathway from natural microbial resources to the development of precision functional products remains unclear. This review focuses on the central theme of synergy between the substrate and microbial modules, including the overall effects of vermicompost on nitrogen, mechanisms analyzed at the omics level, the mining of functional microbial resources and the design of synthetic microbial communities, as well as field-based targeted regulation and validation of effects, to clarify the core principles of synergistic nitrogen promotion and pathways for technological advancement. We further situate these mechanisms within the broader context of crop root plasticity and plant microbial symbioses, linking vermicompost research to current advances in nutrient acquisition. This review provides a systematic theoretical framework for in-depth research into the mechanisms by which vermicompost promotes nitrogen and for the development of green biofertilizers.

## 1 Introduction

The large-scale application of chemical nitrogen fertilizers is a key factor in ensuring high yields in modern agriculture; however, the contradiction between their excessive use and low utilization efficiency is becoming increasingly prominent. The resulting soil degradation and environmental issues are key factors limiting the sustainable development of agriculture (Jiang et al., 2024; Food and Agriculture Organization, 2025). The development of green inputs that can replace or supplement chemical nitrogen fertilizers has become one of the core issues in the green transformation of agriculture. “Green inputs” are defined as low-impact agricultural alternatives or supplements to chemical fertilizers, such as organic fertilizers, biofertilizers, and microbial inoculants. These inputs sustain crop yields while mitigating nitrogen losses and greenhouse-gas emissions. The low nitrogen-use efficiency of chemical fertilizers (typically 30%–50%) means that a substantial fraction of applied nitrogen is lost through leaching, ammonia volatilization, and denitrification, and denitrification of surplus nitrogen emits nitrous oxide (N_2_O), a potent greenhouse gas that also contributes to stratospheric ozone depletion. Climate-smart fertilizer technologies, including slow- and controlled-release fertilizers and organic amendments, have been developed to mitigate these emissions (Mohd Zuki et al., 2020; Geelani et al., 2026). Vermicompost, the complete industrial vermicomposting product composed of earthworm castings and partially decomposed organic residues, is the end product of the synergistic conversion of organic waste by earthworms and microorganisms. It is rich in organic matter, possesses a stable humus structure, and carries a highly active microbial community. It can significantly increase soil nitrogen storage capacity and promote plant nitrogen acquisition by crops (Maji et al., 2017; Vidal et al., 2019; Nonthapa et al., 2024). Compared with chemical nitrogen fertilizers and conventional organic fertilizers, vermicompost has a more stable aggregate structure and a higher degree of humification, and it carries a large number of functional microorganisms that have been filtered through the earthworms’ digestive tracts. These multiple physicochemical and biological characteristics endow it with superior nitrogen regulation potential. For the purposes of this review, the term “vermicompost” specifically denotes the stabilized solid material generated via earthworm-mediated organic waste processing. Related earthworm-derived products, including fresh earthworm casts, vermicompost tea, vermiwash, liquid extracts, and leachates, exhibit distinct chemical and microbial profiles and are therefore not considered equivalent herein.

Understanding of the nitrogen-promoting effects of vermicompost is gradually evolving from a focus on slow release of nutrients to a microbial-driven perspective. Previous studies largely attributed these effects to the slow release of nutrients from humified organic matter (Atiyeh et al., 2002; Domínguez et al., 2010); however, with the advancement of metagenomics technologies, the contribution of the abundant functional microorganisms carried by vermicompost to nitrogen transformation has gradually been revealed (Chen and Whalen, 2016; Wang et al., 2018). Therefore, research on the nitrogen-promoting effects of vermicompost must view it as a composite system consisting of two major functional modules, the substrate and the microorganisms, and deconstruct and analyze the functions of these two modules to reveal the essence of their effects.

Current understanding of the nitrogen-promoting effect of vermicompost largely remains at the level of verifying overall fertilizer efficacy, while a systematic analytical framework for the independent contributions and synergistic mechanisms of vermicompost’s physicochemical substrate and endogenous microbial communities has yet to be established. At the same time, the pathways for mining and constructing precise functional modules from the natural microbial resources in vermicompost remain unclear, limiting the efficient utilization of this resource and product upgrading. Unlike existing reviews, which largely focus on the overall fertilizing efficacy of vermicompost or the characteristics of a single microbial community (Vuković et al., 2021; Ducasse et al., 2022; Amaya-Gómez et al., 2025), this review focuses on constructing an analytical framework from the perspective of synergy between the substrate and microbial modules. It critically synthesizes research progress across the entire chain, from the physicochemical properties of vermicompost to functional microbial resources, and identifies current research gaps and future directions for breakthroughs in the field application of synthetic microbial communities (SynComs).

## 2 The overall effect of vermicompost on plant nitrogen acquisition

### 2.1 Enhancing soil nitrogen pools and plant nitrogen acquisition

When vermicompost is applied to the soil, it can significantly increase both the total nitrogen content and the available nitrogen in the soil. Earthworm castings refer to high-nitrogen excreta formed when earthworms consume low-nitrogen organic waste, such as crop straw and livestock manure, which are then digested in their intestines and converted through the synergistic action of endogenous microorganisms (Meena et al., 2024; Velásquez-Chávez et al., 2025). Earthworm castings’ total nitrogen content is significantly higher than that of the feeding substrate and surrounding soil, thereby increasing soil nitrogen input at the source (Jouquet et al., 2008; Vos et al., 2019). Vermicompost supplies nitrogen as both an organic reservoir and mineral forms. Mineral pools consist primarily of NH_4_^+^ and NO_3_^-^. The mineral fraction is directly available to plants, whereas the organic fraction is released gradually through microbial mineralization, and this process is accelerated by the abundant nitrogen-cycling microorganisms carried by vermicompost (Jiménez et al., 2001; European Commission, 2010). The inorganic nitrogen is primarily generated through the breakdown of complex organic nitrogen in the earthworm’s gut and subsequent mineralization by microorganisms in the earthworm castings, which can significantly increase the supply of available nitrogen in the soil (Coq et al., 2007); the substrate-level mineralization dynamics of vermicompost are examined in detail in Section 3.1 and 3.2. Ammonium is acquired mainly through AMT-type ammonium transporters, whereas nitrate is taken up by NRT-type nitrate transporters; the nitrate transceptor NRT1.1 additionally senses nitrate availability and modulates root architecture (Bouguyon et al., 2015). Absorbed nitrogen is assimilated via the glutamine synthetase–glutamate synthase (GS-GOGAT) pathway, and nitrate transporter NRT1.1B has further been linked to root microbiota composition and nitrogen use in field-grown rice (Zhang et al., 2019). These plant-side uptake systems are complemented by vermicompost-borne microorganisms that promote root growth and plant nitrogen acquisition, completing the substrate–microbial–root axis of nitrogen acquisition (**Figure 1**). Relevant pot experiments indicated that application of vermicompost could increase soil total-nitrogen content. On a 100% dry-matter basis, values rose from 0.26% in the vermicompost-free control soil to 2.12% following vermicompost amendment, representing a 715% increase. Ammonium-N and nitrate-N reached 28.5 mg/kg and 315.5 mg/kg, compared with 5.8 mg/kg and 12.9 mg/kg for the control, respectively (Kováčik et al., 2026). The extent of these increases is influenced by factors such as earthworm species, feeding substrate, and composting conditions; actual effects must be evaluated in the context of specific conditions. Therefore, soil treated with vermicompost can provide crops with more abundant and sustained nitrogen nutrition, leading to significant improvements in crop dry matter accumulation, nitrogen uptake efficiency, and leaf nitrogen content (Van Groenigen et al., 2014).

**Figure 1:**
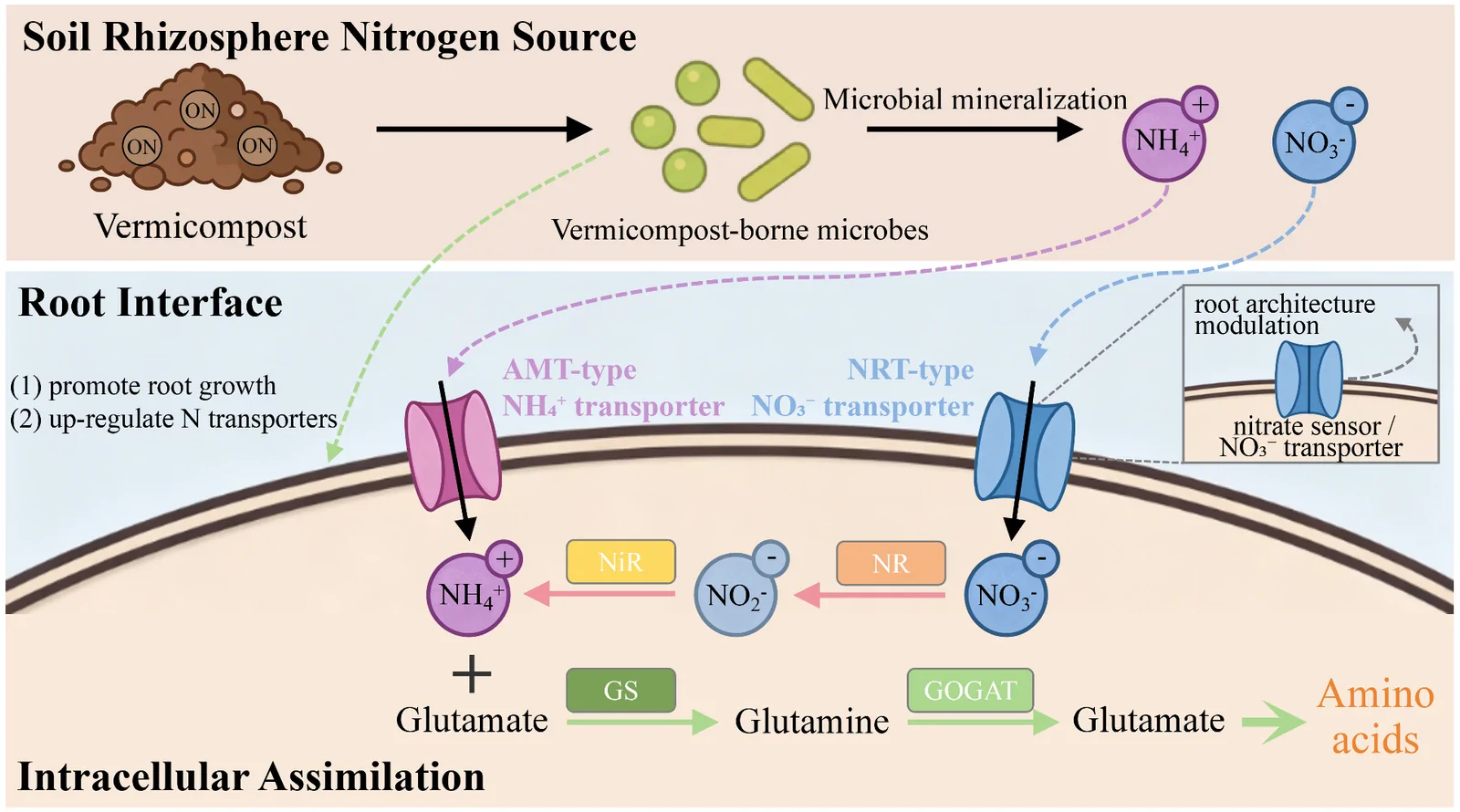
Schematic diagram illustrating how crop roots acquire the two nitrogen forms supplied by vermicompost. Organic nitrogen is mineralized in the rhizosphere by vermicompost-borne microorganisms; ammonium (NH_4_^+^) and nitrate (NO_3_^-^) are then taken up across the root plasma membrane through AMT-type ammonium transporters and NRT-type nitrate transporters, respectively, and assimilated via the glutamine synthetase-glutamate synthase (GS-GOGAT) pathway; nitrate is first reduced to ammonium via nitrate reductase (NR) and nitrite reductase (NiR) before entering this pathway. Vermicompost-derived microorganisms also promote root growth and nitrogen transporter expression, expanding the crop's nitrogen acquisition capacity.

Beyond raising the soil nitrogen pool, vermicompost consistently improves crop nitrogen acquisition in quantitative terms. In continuous-cropping pepper, vermicompost increased nitrogen, phosphorus, and potassium accumulation by 17–58% and improved fertilizer use efficiency relative to farmer practice (Zhang et al., 2023). A systematic review of integrated nutrient management reached a similar conclusion, reporting that partial substitution of inorganic nitrogen with vermicompost consistently enhanced crop nitrogen acquisition across both legumes and cereals (Sande et al., 2024). Together with the synergistic effect on plant nitrogen acquisition under reduced mineral nitrogen rates discussed in Section 2.3 (Arif et al., 2026) and the root morphological adjustments described in Section 3.4 (**Table 1**), these findings indicate that the plant-side benefits of vermicompost extend beyond mechanism to measurable, yield-determining nitrogen capture, albeit with magnitudes that remain context-dependent.

**Table 1:** Reported effects of vermicompost amendments on root architecture and plant symbioses.

Treatment	Observed phenotype	References
Vermicompost application	Increased lateral-root and root-hair proliferation; expanded root foraging volume	(Sarkar et al., 2023; Zhang et al., 2024)
Humic-acid-rich vermicompost	Enhanced root nodulation and arbuscular mycorrhizal colonization in Pisum sativum	(Maji et al., 2017)
Vermicompost combined with arbuscular mycorrhizal fungi	Improved lemongrass growth relative to either treatment alone	(León-Anzueto et al., 2011)

### 2.2 Enriching beneficial microorganisms and activating the nitrogen cycle

As an organic fertilizer, vermicompost can carry and introduce beneficial functional microorganisms related to the nitrogen cycle into the soil. The earthworm’s gut is a natural system for the selection and enrichment of microorganisms; as organic matter passes through the digestive tract, specific microbial groups are selectively enriched and excreted with the earthworm castings (Mirleau et al., 2024). Once vermicompost enters the soil, these intestinal microorganisms can colonize the new microenvironment and establish symbiotic relationships with indigenous microorganisms, thereby altering the community structure and function of the soil microbiome (Du et al., 2026; Karine et al., 2024; Tan et al., 2021; (Yang et al., 2024a). Consequently, vermicompost forms microbial hotspots, with microbial biomass and metabolic activity significantly higher than in ordinary farmland soil.

Molecular biology techniques such as metagenomics have confirmed that vermicompost is enriched with diverse microorganisms engaged in the soil nitrogen cycle. Rather than a single linear “nitrogen-promoting” sequence, these processes are more accurately described across five functional dimensions. The first is nitrogen transformation, which includes mineralization-ammonification and nitrification that convert organic N into plant-available forms. The second is nitrogen availability, referring to the ammonium and nitrate pool accessible to plants. The third is nitrogen retention, representing nitrogen stabilization within soil organic matter and microbial biomass to reduce nitrogen loss. The fourth is nitrogen loss, driven by denitrification, leaching and volatilization that deplete plant-available N while releasing N_2_O and N_2_. The final dimension is plant acquisition, defined as root uptake of the available nitrogen pool (Wang et al., 2018; Li et al., 2020). In terms of biological nitrogen fixation, genera such as *Rhizobium*, *Azotobacter*, *Azospirillum*, and *Bradyrhizobium* can convert atmospheric nitrogen gas (N_2_) into ammonia nitrogen (NH_3_), thereby supplementing exogenous nitrogen inputs to the soil (Li et al., 2020; Rabara et al., 2023; Soltan et al., 2022). Ammonification, on the other hand, is primarily driven by saprophytic microorganisms such as the genera *Bacillus* and *Pseudomonas*, which mineralize organic nitrogen into ammonium nitrogen (NH_4_^+^), providing a substrate for subsequent nitrification (Mapile and Obusan, 2020). Nitrification is carried out by two types of functional microbial communities. Ammonia oxidation is performed by ammonia-oxidizing archaea (AOA) and ammonia-oxidizing bacteria (AOB, e.g., *Nitrosomonas* and *Nitrosococcus*), whereas a subset of *Nitrospira* termed complete ammonia oxidizers (comammox) can oxidize ammonia to nitrate within a single organism; nitrite-oxidizing bacteria, such as *Nitrospira* and *Nitrobacter*, further oxidize NO_2_^-^ to nitrate (NO_3_^-^) (Prosser and Nicol, 2012; Daims et al., 2015; Van Kessel et al., 2015; Nechayeva et al., 2021; Mubaraq et al., 2024). Denitrification is carried out by bacteria such as *Pseudomonas* and *Bradyrhizobium*, which gradually reduce NO_3_^-^ to nitrous oxide (N_2_O) or nitrogen gas (N_2_) (Pishgar et al., 2021). Notably, the activity of denitrifying bacteria is limited in well-aerated upland soils, resulting in relatively limited actual nitrogen loss. Among these, nitrogen-fixing, ammonifying, and nitrifying populations collectively enhance the plant-available N supply, whereas denitrifiers constitute a distinct loss pathway whose activity is constrained rather than promoted; the net effect of these diverse functional groups is to drive multistep nitrogen transformation while limiting nitrogen loss, thereby activating soil nitrogen cycling.

### 2.3 Differences in nitrogen allocation and utilization characteristics under different fertilization regimens

Vermicompost offers distinct advantages in optimizing soil nitrogen distribution and improving crop nitrogen-use efficiency; there are significant differences in nitrogen transformation pathways among different fertilization regimes (**Figure 2**). Although the sole application of chemical nitrogen fertilizers increases crop yields by 40%-60%, crop nitrogen-use efficiency remains as low as 30%-50%. Unabsorbed nitrogen is either fixed in soil organic matter, retained as residual inorganic nitrogen, or lost to the surrounding environment through leaching, volatilization, or denitrification, which can easily lead to resource waste and non-point source pollution (Leicht, 2024; Mohd Zuki et al., 2020). The complementarity of these two fertilizers is the basis of their synergistic effect: the chemical fertilizer provides readily available inorganic nitrogen to meet the crop’s immediate demand, whereas vermicompost supplies slow-release organic nitrogen together with a physicochemical buffering capacity and a functional microbial community.

**Figure 2:**
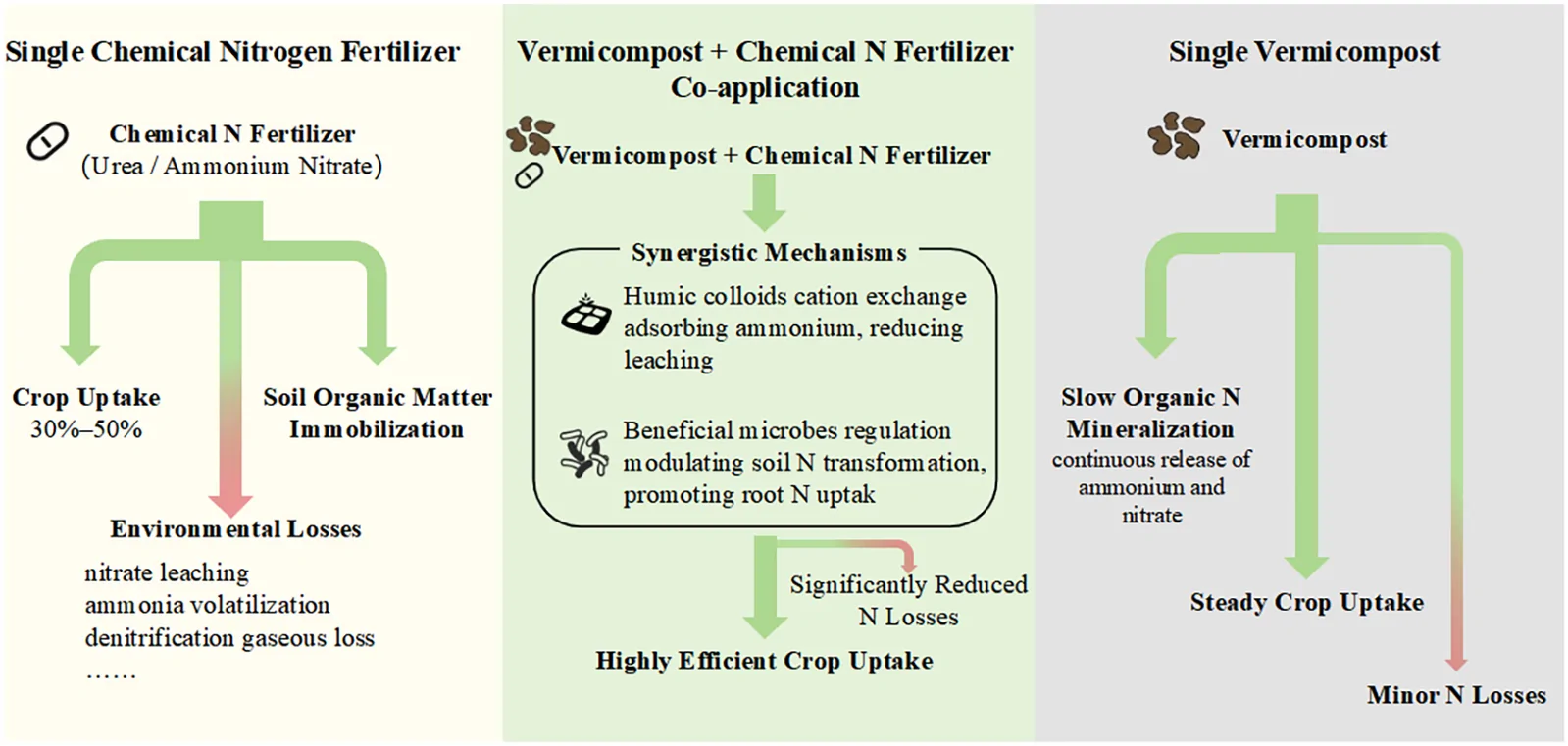
Schematic diagram comparing soil nitrogen fate and utilization efficiency under different fertilization methods. The left panel represents sole chemical nitrogen fertilizer supplied as urea or ammonium nitrate. Only 30% to 50% of applied nitrogen is taken up by crops, while part of the nitrogen becomes immobilized within soil organic matter and substantial nitrogen is lost through nitrate leaching, ammonia volatilization and denitrification (primarily in localized anaerobic microsites). The right panel illustrates sole vermicompost application. Slow mineralization of organic nitrogen continuously releases ammonium and nitrate to sustain steady crop uptake with limited nitrogen loss. The middle light-green panel shows combined application of vermicompost and chemical nitrogen fertilizer. Two complementary synergistic effects are presented within the box. Humic colloids retain ammonium via cation exchange to lower leaching risks, and beneficial soil microbes regulate nitrogen transformation processes to facilitate root nitrogen uptake. Overall, co-application outperforms either sole application by combining rapid nutrient supply with reduced nitrogen losses.

Seen from the perspective of a whole-system nitrogen balance, the benefit of any fertilization regime lies in how the applied nitrogen is partitioned among crop recovery, soil retention, and losses, rather than in the nitrogen-use efficiency ratio alone. A nitrogen balance tallies all nitrogen inputs, including chemical fertilizer, vermicompost, and biologically fixed nitrogen, against plant uptake, gaseous and leaching losses, and residual soil nitrogen, so that true improvements in recovery can be separated from shifts among loss pathways (Mohd Zuki et al., 2020; Leicht, 2024). The slow-release, microbe-mediated supply of vermicompost is best understood as shifting this partition toward greater plant recovery and lower loss, a point developed further in the field assessments below.

In contrast, the sole application of vermicompost represents a slow-release organic nitrogen supply mode. Its nutrients coexist in both organic and chelated forms, releasing slowly and persistently, which better matches crop nitrogen requirements throughout the entire growth period, keeping overall nitrogen loss at a low level (Fikru and Fikreyohannes, 2018; Hoque et al., 2022). When vermicompost is applied in combination with chemical nitrogen fertilizers, the two can produce complementary and synergistic effects. For example, field experiments have shown that reducing the mineral nitrogen rate alone lowers maize yield, whereas combining the same reduced nitrogen rate with vermicompost maintains grain yield comparable to the full-rate fertilizer treatment and raises total plant nitrogen acquisition to 112.6 kg/ha, effectively offsetting the yield penalty of nitrogen reduction (Arif et al., 2026). This indicates that vermicompost can significantly improve the utilization efficiency of chemical nitrogen fertilizers and reduce the risk of nitrogen loss. This synergistic effect is achieved primarily through two pathways. The first is a physicochemical pathway, in which humic colloids in vermicompost adsorb ammonium nitrogen via cation exchange to reduce leaching losses (Pereira and Arruda, 2003; Liu et al., 2020); second, a biological pathway, whereby beneficial microorganisms introduced by vermicompost regulate nitrogen transformation processes in the rhizosphere and promote plant nitrogen acquisition by plant roots (Omokaro, 2024; Canning, 2026). The combined action of these two mechanisms significantly reduces total nitrogen loss in the system, thereby realizing the nitrogen-promoting potential of vermicompost. Nevertheless, these benefits are context dependent: vermicompost altered soil microbial communities and increased nitrate leaching under tropical sugarcane, a reminder that outcomes vary with crop, climate, and management and that potential trade-offs must be weighed alongside the gains (Canning, 2026).

Chemical fertilizers also influence soil microorganisms. Long-term nitrogen fertilizer inputs modulate soil microbial biomass and extracellular enzyme activities, with responses that differ between upland and paddy soils (Ali et al., 2021). In contrast, vermicompost amendment enriches functional microbial communities and enhances enzymatic activity (Aechra et al., 2021; Yang et al., 2024b), helping to maintain a more diverse and active soil microbiome. These contrasting effects at the microbial level provide an additional rationale for substituting or supplementing chemical nitrogen fertilizers with vermicompost.

## 3 Module-based analysis of the nitrogen-promoting effect: substrate vs. microorganisms

### 3.1 Substrate-based nitrogen retention and supply functions

The nitrogen-promoting effect of vermicompost is based on the substrate’s dual function as both a nutrient reservoir and a soil conditioner. Earthworm casting substrates contain three core chemical components: organic matter, humus, and mineral nutrients. Among these, organic matter primarily provides a slow-release source of organic nitrogen; humus, composed mainly of humic acid and fulvic acid, improves soil physicochemical properties; and mineral nutrients, dominated by nitrogen, phosphorus, and potassium, are also rich in calcium, magnesium, sulfur, and various trace elements. Overall, the nutrient availability of this substrate is significantly higher than that of ordinary soil and uncomposted organic matter (Sinha et al., 2009; Rakkini et al., 2025). In terms of nitrogen supply characteristics, the nitrogen in vermicompost consists primarily of organic nitrogen bound to humus, which is released at a steady rate. It can be gradually mineralized by soil microbial activity to form ammonium and nitrate nitrogen, maintaining a stable nitrogen supply throughout the entire crop growth cycle. Mineralization proceeds through ammonification and subsequent nitrification, with the rate and extent of net nitrogen release depending on substrate quality and the activity of the mineralizing microbial community, as demonstrated by incubation studies on soils amended with different proportions of vermicompost (Walpola and Wanniarachchi, 2009; Askari et al., 2020). This stands in stark contrast to the characteristics of chemical nitrogen fertilizers, which are fast-acting but prone to leaching (Askari et al., 2020). The abundant humic acid and fulvic acid colloids in vermicompost possess high specific surface areas and cation exchange capacities. Through electrostatic interactions, they adsorb positively charged ammonium ions, thereby establishing a stable nutrient buffer reservoir within the soil. This significantly reduces nitrogen leaching losses caused by water infiltration while simultaneously enhancing soil nutrient retention capacity and long-term nitrogen supply stability (León-Anzueto et al., 2011; Yildirim et al., 2021; Lastiri-Hernández et al., 2023). These dual properties, slow-release nitrogen supply and colloidal nitrogen fixation, together constitute the core nitrogen-promoting foundation of vermicompost at the substrate level.

### 3.2 The driving effect of microbial nitrogen transformation

The nitrogen-promoting effect of vermicompost depends on the direct role of its microbial community in nitrogen transformation (**Figure 3**). The functionally diverse, highly active microbial community in vermicompost drives all stages of nitrogen transformation ranging from organic nitrogen decomposition to inorganic nitrogen formation and acts as one of the key mediators of this effect, functioning in concert with the substrate effects detailed in Section 3.1 (Walpola and Wanniarachchi, 2009; Jeevapriya and Kanimozhi, 2024; Amaya-Gómez et al., 2025). The enrichment and activation of this microbial community are achieved synergistically through dual pathways, both within and outside the earthworm’s body. The in vivo pathway, known as the gut-associated process (GAP), involves the selective activation and proliferation of indigenous microorganisms from the diet under conditions specific to the earthworm’s gut, such as a microanaerobic environment, enzyme activity hotspots, and moisture and soluble carbon carried by intestinal mucus, which are ultimately excreted with earthworm castings (Devliegher and Verstraete, 1995; Drake and Horn, 2007; Thakuria et al., 2010; Wüst et al., 2011; Jin et al., 2022). Once earthworm castings are excreted via the in vivo pathway and enter the soil, they become the core component of the ex vivo pathway. The ex vivo pathway is primarily manifested through the drilosphere, a microhabitat jointly created by earthworm castings, mucin glycoproteins, deposits, readily degradable carbon and nitrogen, and earthworm burrowing activity. Mucin glycoproteins and digestive enzymes reawaken dormant indigenous microorganisms in the soil, transitioning them to an active state and enriching the microbial communities important for soil nitrogen cycling; this phenomenon is also known as the “Sleeping Beauty Paradox” (Taheri, 2018; Ahmed and Al-Mutairi, 2022). The continuous cycling through both in vivo and ex vivo pathways maintains the microbial biomass nitrogen in earthworm castings at a high level, constituting a core regulatory node for the interconversion of organic and inorganic nitrogen.

**Figure 3:**
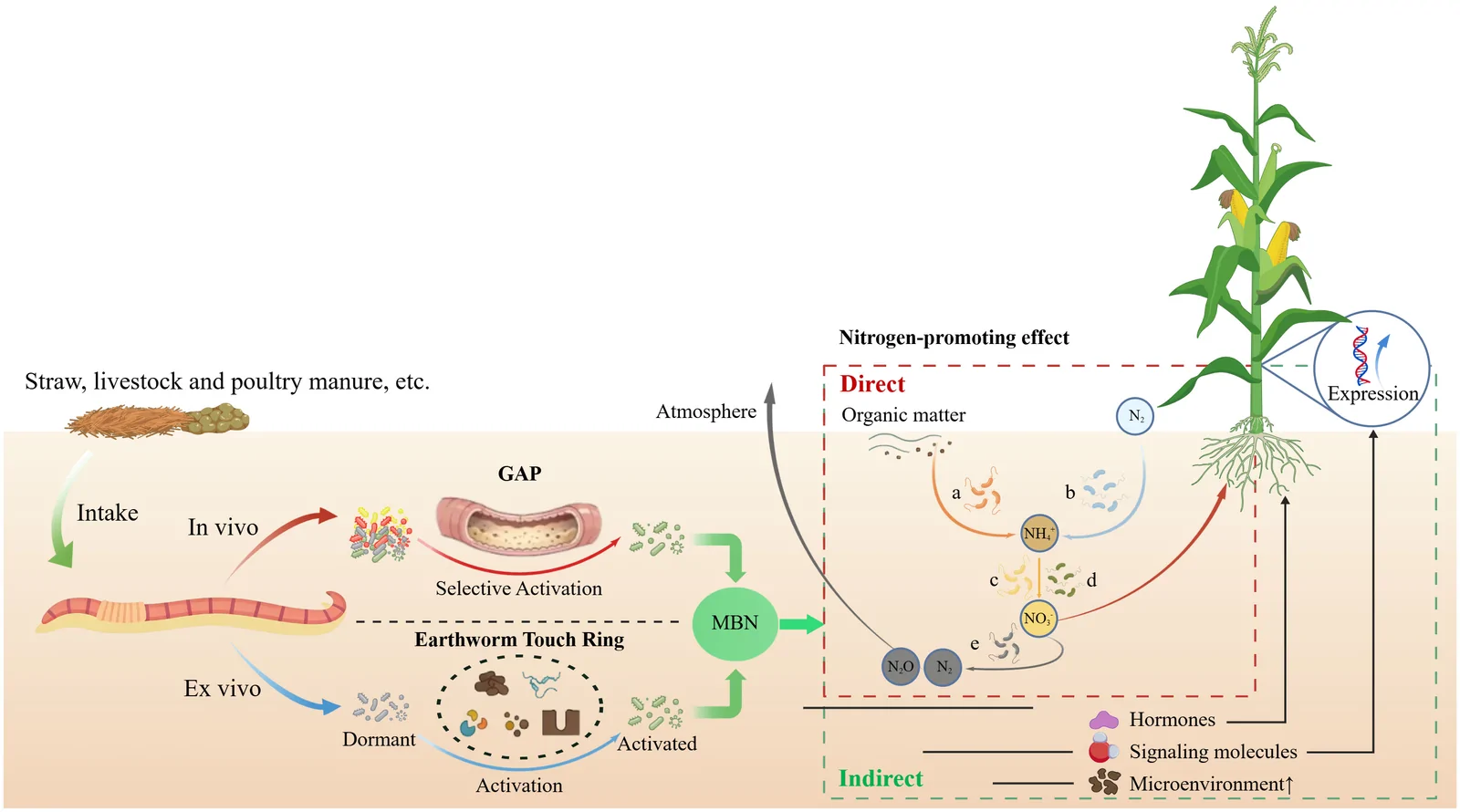
Schematic diagram illustrating nitrogen transformation mediated by vermicompost microorganisms. Two linked pathways including the in vivo gut-associated process and ex vivo drilosphere microhabitat jointly assemble functional microbial communities to form the mineral-beneficial nitrogen pool named MBN. MBN mediates maize nitrogen acquisition through two independent regulatory branches separated by dashed frames. The red-framed direct branch relies on four functional microbial populations marked a to d to drive nitrogen transformation through functionally distinct guilds. Ammonifiers decompose organic matter to produce ammonium, and nitrogen-fixing microbes convert atmospheric nitrogen into ammonium. Ammonia oxidation is performed by ammonia-oxidizing archaea (AOA) and bacteria (AOB), and nitrite oxidation by nitrite-oxidizing bacteria such as Nitrospira (including comammox Nitrospira that oxidize ammonia to nitrate in a single organism). Denitrification, leaching, and volatilization constitute parallel nitrogen-loss pathways that deplete plant-available nitrogen and therefore are shown outside the direct branch (see Section 3.2). The green-framed indirect branch covers three independent regulatory modes. Microbial phytohormones, soluble signal compounds and modified rhizosphere microhabitats each upregulate crop nitrogen transporter genes and expand root nutrient acquisition capacity. Created with BioGDP.com (Jiang et al., 2025).

In short, vermicompost-derived microorganisms directly participate in soil nitrogen transformation through functionally distinct groups that fall into two categories: nitrogen-acquisition processes (nitrogen fixation, ammonification/mineralization, nitrification, and plant uptake) and nitrogen-loss processes (denitrification, leaching, and volatilization). Rather than a single linear chain, these groups operate across separate compartments of the soil nitrogen cycle, with denitrification representing a loss pathway rather than a step toward plant nitrogen acquisition. The promotion of soil nitrogen cycling by earthworm casting-derived microorganisms can be divided into two basic pathways: direct and indirect. The direct pathway is primarily driven by the diverse nitrogen-cycling microorganisms they enrich (Walpola and Wanniarachchi, 2009; Jeevapriya and Kanimozhi, 2024; Amaya-Gómez et al., 2025). Indirect pathways manifest as the regulation of plant nitrogen acquisition by the metabolic activities of these microorganisms; existing studies have revealed several mechanisms of action from the perspectives of gene expression, root growth, and the rhizosphere microenvironment. As established in Section 2.2 (Li et al., 2025; Xue et al., 2022; Joshi et al., 2015; Spieck and Bock, 2005), the nitrogen-acquisition limb of this pathway encompasses nitrogen fixation, ammonification, and nitrification, which together render nitrogen plant-available, whereas denitrification, leaching, and volatilization constitute parallel loss pathways that deplete plant-available nitrogen; the present section therefore concentrates on the novel dual-pathway mechanism (in vivo gut-associated process and ex vivo drilosphere) by which these communities are selectively enriched and activated (**Figure 3**). Regarding gene expression regulation, bioactive substances such as microbial signal molecules can, in general plant-microbe systems, participate in regulating the expression of genes related to plant nitrogen acquisition; whether vermicompost-borne consortia exert this effect through the same pathways is not yet directly demonstrated in vermicompost systems. For example, a vermicompost-derived consortium was reported to upregulate a tomato high-affinity nitrate transporter gene, though the generality of this finding across vermicompost systems requires confirmation (Garcia et al., 2024). At the systemic level, nitrate perception by the NRT1.1 transceptor integrates local and systemic nitrogen signals (Bouguyon et al., 2015), while CEP peptides coordinate shoot nitrogen demand with root foraging and nodulation (Taleski et al., 2018); notably, the NRT1.1B allele shapes root microbiota composition and thereby rice nitrogen-use efficiency (Zhang et al., 2019), suggesting that vermicompost-derived microbes may interface with this signaling axis. More broadly, root system architecture exhibits pronounced developmental plasticity under nutrient deficiency, enabling plants to reposition foraging effort toward localized nutrient patches (Gruber et al., 2013; Giehl and Von Wiren, 2014), a strategy that microbial inputs can modulate, although direct evidence specifically involving vermicompost-derived microbes remains limited (Oldroyd and Leyser, 2020). In terms of improving the rhizosphere microenvironment, microbial metabolic activity can alter the physical and biological properties of rhizosphere soil; for example, it reshapes bacterial community structure and improves the microstructure of soil aggregates, thereby providing conditions for the colonization and metabolism of functional microbial communities involved in the nitrogen cycle (Liu et al., 2024). In summary, microorganisms derived from vermicompost synergistically enhance plant nitrogen acquisition and utilization through two mechanisms: direct transformation and indirect regulation, the phenotypic expression of this indirect hormonal regulation is integrated with the substrate–microbial synergy discussed in Section 3.3. Regarding root growth promotion, microbial metabolites with phytohormone-like activity can modulate root architecture. Indoleacetic acid (IAA), a canonical auxin, is well documented to promote lateral root and root hair development (Sarkar et al., 2023). By contrast, jasmonic acid (JA) and salicylic acid (SA) influence root development in a manner strongly dependent on hormone concentration, developmental stage, and stress status, and should not be generalized as root-growth-promoting signals; for instance, in canola JA increased lateral but shortened primary root growth while SA effects varied with salinity (Farhangi-Abriz et al., 2019), and SA-induced adventitious rooting acts largely by raising free IAA (Dong et al., 2020). Their roles in vermicompost-driven root plasticity remain to be directly established.

Most of the aforementioned mechanisms have been elucidated through controlled experiments and microcosm systems; however, their extrapolation to field environments remains subject to scale differences. The colonization efficiency of earthworm casting-derived microorganisms after application to soil is jointly regulated by factors such as competition from indigenous microorganisms, carbon source availability, and soil physicochemical properties (Du et al., 2026; Ma et al., 2026); their *in situ* colonization patterns and functional stability under field conditions still require systematic evaluation.

### 3.3 Synergistic nitrogen-activation mechanisms involving substrates and microorganisms

Building on the substrate nitrogen-supply foundation established in Section 3.1, the nitrogen-promoting effect of vermicompost is not simply the sum of the substrate and microbial functions; rather, the deep synergistic interaction between the two is the core mechanism by which its nitrogen-promoting activity surpasses that of conventional organic fertilizers with equivalent nutrient content. As a physicochemical foundation, the substrate provides support for microorganisms in three ways: first, by supplying carbon substrates, vermicompost has a high organic matter content and bioavailable carbon components, which can continuously provide carbon sources and energy for the metabolic activities of functional microorganisms; second, by constructing aggregate microhabitats, which increase soil porosity and provide physical shelter for microbial colonization; third, by environmental buffering capacity, humus possesses redox activity, which can buffer soil pH fluctuations within a certain range and participate in the regulation of redox balance (Munnoli and Bhosle, 2011; Lu et al., 2024). Microorganisms, acting as the engine of biotransformation, exert a reciprocal effect on the substrate by secreting extracellular enzymes to activate hard-to-utilize organic nitrogen, drive the transformation of nitrogen in various forms, and advance the humification process, thereby enhancing the bioavailability of nitrogen in the substrate (Adrian et al., 2016; Ali et al., 2021).

The nitrogen-promoting contributions of the two components exhibit distinct temporal differentiation: in the early stages, the substrate’s regulatory effect predominates, facilitating the initiation of the microbial community; as the incubation period extends, the microbial activation effect continues to amplify and becomes dominant, exhibiting a sequential synergistic pattern of substrate initiation followed by microbial amplification (Chiranjeeb and Prasad, 2020). Currently, there is no unified standard for the quantitative separation of the nitrogen-promoting contributions of these two types of components; at present, the microbial effect and the nutrient effect of the substrate are primarily separated using the sterilization or inhibition control method (Garcia et al., 2024; Javanmardi et al., 2024). However, the strength of the synergistic effect between the two is jointly regulated by raw materials, composting duration, and the soil environment. In the future, a standardized quantitative separation system needs to be established to deepen our understanding of the synergistic nitrogen-promoting mechanism.

### 3.4 Vermicompost, root plasticity, and plant symbioses: state of evidence

As discussed in Section 3.2, vermicompost-derived microorganisms are proposed to regulate root plasticity through phytohormone and nitrate signaling pathways (e.g., NRT1.1 and CEP peptides), by analogy with better-characterized plant-microbe systems; direct experimental evidence for these signaling-mediated effects in vermicompost systems remains limited, and such mechanisms should therefore be regarded as plausible rather than demonstrated. Beyond these direct regulatory mechanisms, the interaction between vermicompost and plant symbiotic systems-particularly arbuscular mycorrhizal (AM) fungi-constitutes another critical, yet underexplored, dimension of nutrient acquisition. The table below maps the documented effects of vermicompost applications on root architecture and symbiotic phenotypes from the existing literature (**Table 1**).

The evidence for a vermicompost-mycorrhiza link, however, remains sparse. Although arbuscular mycorrhizal fungi enhance plant nitrogen and phosphorus uptake through dedicated transporters such as the maize mycorrhiza-specific ammonium transporter ZmAMT3;1 and the *Medicago* phosphate transporter MtPT4, and are sustained by a reciprocal carbon-for-nitrogen exchange, these mechanisms have not been demonstrated in vermicompost systems (Javot et al., 2007; Kiers et al., 2011; Luginbuehl et al., 2017; Hui et al., 2022). The few studies that do connect vermicompost to root symbioses are observational and call for controlled experiments that quantify colonization, transporter expression, and carbon flux (León-Anzueto et al., 2011; Maji et al., 2017). Accordingly, closing this gap-by quantifying colonization, transporter expression, and carbon flux in vermicompost-amended systems-is a high-priority direction for aligning vermicompost research with the symbiotic-exchange theme of this special issue.

Building on these established mechanisms, it is reasonable to speculate that, within vermicompost-amended systems, vermicompost-borne microorganisms and their metabolites may feed into this symbiotic axis: they could modulate root exudation, which in turn conditions AM fungal recruitment and colonization; the established mycorrhizal uptake machinery-the maize ammonium transporter ZmAMT3;1 and the Medicago phosphate transporter MtPT4-would then channel symbiotically acquired nitrogen and phosphorus into the plant, while reciprocal carbon allocation (host-derived fatty acids) closes the exchange loop (Javot et al., 2007; Kiers et al., 2011; Luginbuehl et al., 2017; Hui et al., 2022). This sequence is best regarded as a conceptual model to be tested rather than an established pathway in vermicompost systems, and it dovetails with the substrate-microbial-root framework developed in this review.

## 4 Discovery of functional microbial resources and SynCom design

### 4.1 From vermicompost to the screening of functional strains

The microenvironment within earthworm intestines can selectively enrich and reactivate dormant functional microorganisms, causing vermicompost to naturally contain large quantities of nitrogen-cycling strains adapted to organic substrates. We hypothesize that vermicompost-derived strains, when combined with the vermicompost substrate, may exhibit a more pronounced synergistic nitrogen-promoting effect than strains isolated from the rhizosphere or conventional compost; direct comparative evidence for this hypothesis remains limited, yet such potential makes them plausible candidates for the development of integrated organic fertilizer-microbial agent products. Beyond vermicompost, rhizosphere-derived plant growth-promoting bacteria such as Bacillus subtilis and Pseudomonas fluorescens have been isolated from crop rhizospheres and represent complementary PGPB candidates within the SynCom framework (Jat and Das, 2026). Screening for target functional strains based on vermicompost’ss microbial resources is the cornerstone for elucidating nitrogen-promoting mechanisms and developing biofertilizers. Current research has shifted from investigating community composition to function-oriented strain discovery. Although traditional isolation and culture methods remain fundamental tools in microbiological research, limitations inherent in agar plate culture, such as bias, make it difficult to cultivate the vast majority of environmental microorganisms under conventional laboratory conditions, resulting in significant shortcomings in coverage and efficiency when relying on a single screening approach (Amann et al., 1995). The current mainstream strategy employs targeted isolation and cultivation guided by metagenomic data, which can identify sequences carrying key nitrogen cycle functional genes such as *nifH* and *amoA* from environmental DNA and predict the microbial groups to which they belong. Among these, *nifH* is a characteristic marker gene for diazotrophic (nitrogen-fixing) communities, informing the recovery of nitrogen-fixing taxa; nitrification is instead indexed by amoA, consistent with the gene pair noted above. This provides clear guidance for targeted isolation, allowing for the design of selective culture media and the use of high-throughput screening technologies to specifically isolate target strains (Lewis et al., 2021).

Building on the community-level framework established in Section 2.2, culture-dependent isolation, together with culture-independent molecular identification, has advanced the field from profiling to isolating the actual strains resident in earthworm intestines and vermicompost, and possesses notable capacity for the development of high-quality nitrogen-cycling microbial resources nitrogen-cycling (Wüst et al., 2009; Hussain et al., 2016; Kerketta et al., 2025). For biological nitrogen fixation, culture-based studies have recovered diazotrophic isolates with confirmed nitrogenase activity, including *Kluyvera ascorbata* from the gut and *Klebsiella variicola*, whose diazotrophic identity was validated by *nifH* gene analysis (Hussain et al., 2016; Li et al., 2020). For nitrification, 16S rRNA amplicon sequencing of the earthworm gut has recovered ammonia-oxidizing bacteria, consistent with the nitrifier enrichment documented in Section 2.2 (Li et al., 2020; Singh et al., 2015). For denitrification, metagenomic profiling of the earthworm gut has identified denitrifying taxa such as *Paenibacillus terrae* (Horn et al., 2005). These cultured isolates represent only a fraction of the nitrogen-cycling diversity resident in earthworm systems; in reality, the number of nitrogen-cycling microorganisms isolated and identified from earthworm systems far exceeds this. This continuously rich reservoir of bacterial species provides important experimental material for the development of composite biological agents and the efficient regulation of nitrogen in farmland.

### 4.2 SynCom design strategy and functional module assembly

Constructing synthetic microbial communities based on functional strains derived from vermicompost is a key approach to leveraging the synergistic nitrogen-cycling (nitrogen-promoting) effects (Huang et al., 2023; Shayanthan et al., 2022). The field application of single strains suffers from limitations such as limited functional diversity, poor environmental adaptability, and inconsistent efficacy; in natural environments, microbial nitrogen transformation is achieved collectively through microbial community interaction networks (Compant et al., 2019; Kim et al., 2025). By integrating ecological principles with synthetic biology approaches, rationally pairing and assembling strains to form functionally complementary SynComs can ensure the sustained and efficient expression of microbial functions (Großkopf and Soyer, 2014). To achieve the goal of enhancing nitrogen-use efficiency, the design of SynComs based on vermicompost-derived strains must adhere to the principles of modularity and functional redundancy; the core concept is to assemble a compact and highly efficient nitrogen cycling functional module (De Souza et al., 2020; Kumar Sarker et al., 2026). It should be noted that this four-stage workflow represents a conceptual framework and research roadmap distilled from current literature, rather than a fully matured and field-validated production pipeline; systematic assembly and field validation of vermicompost-derived nitrogen-promoting SynComs remain at an early stage. By synthesizing existing research on vermicompost-derived strains and general principles of SynCom design, the process for creating nitrogen-promoting SynComs can be conceptually organized into four stages. The first stage involves the input of functional strain resources: a library of vermicompost-derived strains can be established through a screening system, stockpiling various functional strains capable of nitrogen fixation, organic nitrogen mineralization, nitrification, and plant growth promotion. The second stage involves the assembly of core functional modules. Focusing on the complete nitrogen cycle, three types of functional units can be constructed using the nitrogen-fixing, mineralizing, and nitrifying genera defined in Section 2.2 and isolated in Section 4.1. Here, biological nitrogen fixation represents one functional module rather than the central mechanism: the nitrogen-fixing unit is anchored by the vermicompost-derived diazotroph Klebsiella (Hussain et al., 2016; Li et al., 2020), the organic nitrogen mineralization unit utilizes protease-producing taxa, and the nitrification unit combines ammonia- and nitrite-oxidizing taxa (Ojeda-Morales et al., 2015; Sidorova and Chechkova, 2021; Quirós et al., 2023). Importantly, available evidence does not establish BNF as the dominant mechanism by which vermicompost promotes nitrogen; mineralization, nitrogen retention, nitrification, rhizosphere modification, and enhanced root uptake contribute substantially as well. The third stage focuses on community optimization and functional expansion. On one hand, in vitro compatibility testing can be conducted to screen for stable coexisting strain combinations, and computational modeling and machine learning algorithms are used to optimize community ratios; on the other hand, growth-promoting and stress-tolerant strains are introduced to construct a dual-function module for nutrient activation and plant growth promotion, achieving synergistic enhancement among the rhizosphere microenvironment, crop hosts, and nutrient cycling (Oyserman et al., 2018; Trivedi et al., 2021; Zheng, 2025; Choi et al., 2026). The fourth stage involves the application and commercialization of the final product. The assembled and optimized SynCom is proposed to exhibit functional stability, strong environmental adaptability, and a high degree of human controllability, and may be formulated into microbial inoculant products to support precision agriculture; these outcomes, however, remain prospective rather than established and await direct evidence from field trials and commercialization (Miao et al., 2026; Niu et al., 2026). Recent studies have shown that isolating functional strains from earthworm intestines and assembling them into SynComs can significantly enhance the performance of target functions. Taking herbicide degradation as an example, after two degradation-capable strains isolated from earthworm intestines were assembled into a SynCom, the degradation rate within 72 hours increased from 50.45%–62.35% for a single strain to 69.38%, and complete mineralization of the target compound was achieved (Ge et al., 2025). Although the aforementioned study focused on herbicide degradation rather than nitrogen transformation, it confirmed the feasibility and synergistic advantages of the technical approach, isolating functional strains from earthworm-associated environments, constructing SynComs, and validating synergistic effects, providing a directly applicable methodological reference for strain screening and module assembly of nitrogen-promoting SynComs derived from vermicompost. Currently, research on constructing nitrogen-promoting SynComs based on vermicompost and validating them in field trials remains relatively limited. In the future, it will be necessary to systematically supplement data on the effects of different strain combinations and establish quantitative evaluation criteria.

## 5 Field implementation of nitrogen-promoting functions: targeted regulation and validation of effects

### 5.1 Production processes and quality control focused on nitrogen enrichment

The production workflow can be framed as a three-stage process: feedstock pretreatment, controlled vermicomposting, and product quality control. The screening of functional strains from vermicompost and the SynCom design provide the microbiological foundation for the precise development of nitrogen-promoting products derived from vermicompost; however, realizing this biological potential requires a standardized production process capable of fully preserving the functional microbial community and active forms of nitrogen. For the large-scale production of vermicompost with nitrogen-promoting functions, the key lies in targeting nitrogen enrichment and the enrichment of functional microbial communities, and achieving stable and controllable nitrogen-promoting activity in the product through precise control of raw material formulations and environmental factors. Industrial earthworm composting is the core process by which earthworms and microorganisms synergistically degrade organic waste; environmental and feedstock parameters directly determine the nitrogen composition of the final product and the activity of nitrogen-cycling microbial communities (Vuković et al., 2021; Nath et al., 2023). During the feedstock pretreatment stage, mixed organic waste must be formulated to maintain a carbon-to-nitrogen ratio of 20:1-25:1. This range optimizes nitrogen concentration efficiency, promotes the proliferation of nitrogen-cycling microbial communities, and ensures sustained nitrogen release. A carbon-to-nitrogen ratio that is too low can increase ammonia volatilization losses, while one that is too high reduces nitrogen enrichment efficiency (Nath et al., 2023). Upon entering the controlled earthworm composting stage, the system temperature is maintained at 15-25 °C, the moisture content is regulated to approximately 60%–80%, and the pH is stabilized at 6.0-8.0. At the same time, adequate aeration is ensured to provide a suitable environment for earthworm activity and the synergistic metabolism of functional microorganisms; the complete treatment cycle lasts approximately 12 weeks (Garg et al., 2006; Robe Elema, 2021; Nath et al., 2023). After passing quality control, the standardized product can be formulated and deployed for field application (Sections 5.2 and 5.3), completing the chain from preparation to commercial deployment.

Upon completion of composting, a multi-indicator quality control system covering chemical, physical, and biological dimensions must be established to test the final product. Chemical indicators are subject to mandatory control; typical parameters include organic matter content, nitrogen, phosphorus, and potassium (N, P_2_O_5_, K_2_O) nutrient levels, pH, carbon-to-nitrogen ratio, and heavy metal content. The typical range for organic matter content is no less than 20%–30%, reflecting the degree of decomposition and soil-improving capacity (Qi, 2012). Nitrogen, phosphorus, and potassium (N, P_2_O_5_, K_2_O) levels vary depending on the raw materials and must be clearly labeled to characterize nutrient supply capacity; pH should be maintained within the range of 6.0-8.0 to ensure nutrient availability; a carbon-to-nitrogen ratio below 20:1 serves as an indicator of decomposition degree; and heavy metal levels must comply with relevant safety limits (Kumar et al., 2013; Zhao, 2015; Nath et al., 2023). Physical indicators relate to storage and transportation characteristics; moisture content is typically required to be no more than 50% to facilitate storage and application, while bulk density and porosity may be optionally declared to reflect the product’s potential to improve soil physical structure (Moreira and Santos, 2012). Biological indicators pertain to the nitrogen-promoting function itself; the abundance of nitrogen cycle-related genes and enzyme activity reflect biological function (Qi, 2012), and pathogen levels must comply with health standards to ensure application safety. Antimicrobial-resistance (AMR) genes should also be considered a biological safety indicator, given that metagenomic surveys have detected diverse antibiotic-resistance genes in earthworm castings (Li et al., 2020). However, at this stage, such biological indicators are mostly used as references for grading, and uniform quantitative standards are still lacking (Kong et al., 2024; Hepşen Türkay, 2025). Standardized control across the entire chain, including raw material ratios, composting environment regulation, and testing of finished product indicators can reliably preserve the nitrogen-promoting properties of vermicompost and support their standardized industrial application. The full list of QC parameters and their rationale is summarized in **Table 2**.

**Table 2:** Proposed quality-control parameters for vermicompost-based products.

Parameter	Category	Significance	Criterion & remarks	Reference
Total N	Chemical	Overall N supply capacity	Must be labeled; typically 1%–2% of dry matter	(Bary et al., 2002)
NH^4+^-N	Chemical	Readily available N fraction	< 500 mg/kg	(Bary et al., 2002)
NO_3_^-^-N	Chemical	Readily available N fraction	200–500 mg/kg; an NH_4_^+^:NO_3_^-^ ratio < 0.16 indicates maturity	(Bernai et al., 1998; Bary et al., 2002)
Organic C	Chemical	Decomposition degree and C source	15%–25% of dry matter	(Standards Australia, 2012)
C:N ratio	Chemical	Decomposition and N stabilization	< 20:1	(Kumar et al., 2013; Zhao, 2015; Nath et al., 2023)
Electrical conductivity	Chemical	Salinity risk	0–4 dS/m; < 2.5 dS/m for potting mixes	(Bary et al., 2002)
pH	Chemical	Nutrient availability	6.0–8.0	(Kumar et al., 2013; Zhao, 2015; Nath et al., 2023)
N, P_2_O_5_, K_2_O and micronutrients	Chemical	Nutrient supply	Must be labeled	(MoA, 2015; Ministry Of Agriculture And Rural Affairs Of The People’s Republic Of China, 2021)
Heavy metals	Chemical	Contamination risk	Comply with applicable safety limits	(Ministry Of Agriculture And Rural Affairs Of The People’s Republic Of China, 2012; Standards Australia, 2012)
Microbial biomass / viable counts	Biological	Functional microbial load	≥ 0.2 × 10^8^ CFU/g	(Ministry Of Agriculture And Rural Affairs Of The People’s Republic Of China, 2012, 2015)
Functional genes (nifH, amoA, etc.)	Biological	N-cycling function	Methods available (e.g., qPCR); product-level limits lacking	(Zhong et al., 2020)
Pathogens	Biological	Application safety	Comply with health standards	(Ministry Of Agriculture And Rural Affairs Of The People’s Republic Of China, 2012)
AMR genes	Biological	Resistance risk	Emerging concern; standardized QC criteria under development	(Tian et al., 2021)
N-mineralization / nitrification activity	Biological	N transformation function	Methods available; product-level limits lacking	(International Organization For Standardization, 2012)
Shelf stability	Physical	Efficacy during storage	≥ 6 months for carrier-based products;1.5–2 years for liquid formulations	(International Organization For Standardization, 2012; Sakpirom et al., 2021; Allouzi et al., 2022)

The listed values are indicative benchmarks compiled from multiple national and international standards (e.g., Standards Australia, ISO, and Chinese Ministry of Agriculture guidelines) and peer-reviewed studies, and are not universal regulatory limits; each parameter should be interpreted within the regulatory context of its cited source. parameters without established product-level limits are indicated as such.

### 5.2 Field application strategies for maximizing the nitrogen-promoting effect

The primary objective of applying vermicompost and derived synthetic microbial community products in the field is to leverage the synergistic effects of nitrogen retention by the substrate and nitrogen-promoting microbial activity. Application strategies can be tailored based on product form, crop type, and soil conditions. Earthworm casting products primarily come in two forms: solid vermicompost and liquid extracts. Solid vermicompost is typically applied as a base fertilizer before sowing or transplanting. Broadcast application followed by tillage can comprehensively increase the nitrogen pool and microbial population in the plow layer, while band or hole application can create microenvironments enriched with nutrients and microorganisms in the crop rhizosphere (Qi, 2012; Hepşen Türkay, 2025). In actual production, depending on soil fertility and crop nitrogen requirements, vermicompost can replace 20%–50% of the nitrogen in chemical fertilizers, balancing the need for rapid nitrogen supply during the seedling stage with the need for slow-release nitrogen supply in later stages (Molla et al., 2019; Ebrahimi et al., 2020; Habtamu et al., 2024). Additionally, vermicompost possesses excellent physicochemical properties and can be used alone or blended with peat moss and perlite to create seedling growing media, making them suitable for cultivating vegetable and flower seedlings (Olaria et al., 2016; Tavali and Uz, 2025). Liquid earthworm casting extracts can rapidly replenish nutrients and beneficial microorganisms through root drenching or foliar spraying (Abdel Salam and Roshdy, 2022; Hamed et al., 2026). Within this liquid category, vermicompost tea, vermiwash, and leachates differ in their production method, nutrient concentration, and microbial load; their distinct properties should be considered when selecting a product for a specific purpose (as defined in the Introduction). Synthetic microbial community formulations prepared using vermicompost strains are available in various forms, including liquids, powders, and granules. They deliver functional microbial communities to the rhizosphere through three targeted methods: seed coating, root drenching during the seedling stage, and mixing with soil granules. Combining seed coating with vermicompost-based growing media further enhances the survival and functional stability of the strains in the field (Manivannan and Daniel, 2009; Mahmood et al., 2016; Rocha et al., 2019). When combined with precision agriculture technologies, the application of these two product types can further enhance their targeting accuracy and nitrogen-promoting effects. In addition, finished vermicompost products can be graded according to maturity, nutrient content, and hygienic indicators, with the corresponding quality control parameters summarized in **Table 2**.

### 5.3 Multidimensional validation and benefit assessment of nitrogen-promoting effects in the field

The nitrogen-promoting effect of vermicompost in the field can be verified in three aspects: crop growth performance, nitrogen-use efficiency, and soil quality improvement; its comprehensive benefits span multiple dimensions, including economic, environmental, and ecological aspects (**Table 3**). In terms of crop production traits, under field trial conditions, maize yield increased by 6.8% following the application of vermicompost, while for wheat, total plant nitrogen acquisition increased from 33.21 kg/ha to 154.33 kg/ha when vermicompost was applied in combination with nitrogen fertilizer nitrogen-use efficiency (Gadisa and Wakgari, 2021; Li et al., 2011). Pot experiments further elucidated the physiological mechanisms underlying these yield increases: rice yields increased by 39% after application of vermicompost; soybean yields increased by 18% to 60% following application of vermicompost derived from different raw materials; and tomato yields increased by 120.8% and fruit vitamin C content increased by 46.4% after foliar spraying with earthworm extract (Dugassa and Worku, 2026; Hamed et al., 2026; Huang et al., 2016). These yield-enhancing effects stem from vermicompost’s long-lasting nitrogen supply capacity, its ability to improve the rhizosphere microenvironment, and its synergistic regulation of functional microbial communities. In terms of nitrogen-use efficiency, field trials have confirmed that the combined application of vermicompost and nitrogen fertilizer can significantly enhance wheat’s nitrogen acquisition capacity, enabling a reduction in chemical fertilizer use while maintaining stable yields and simultaneously lowering production costs and the risk of non-point source pollution nitrogen-use efficiency (Gadisa and Wakgari, 2021). Long-term, continuous application of vermicompost also yields sustained ecological benefits, manifested in the continuous increase in soil organic matter, optimization of soil aggregate structure, and enhanced abundance of nitrogen-cycling microorganisms and enzymatic activity, thereby steadily fostering the soil’s intrinsic nitrogen-supplying capacity (Aechra et al., 2021; Amjadi et al., 2021; Yang et al., 2024b). A comprehensive assessment of the application value of vermicompost in promoting nitrogen management must integrate three types of benefits: economic output, environmental emissions reduction, and soil fertility enhancement, to fully reflect their potential in regulating nitrogen reduction in sustainable agriculture.

**Table 3:** Effects of field and pot application of earthworm-derived materials on yields and nitrogen-use efficiency of selected crops.

Crops	Material	Application method	Research conditions	Performance metrics	Degree of efficiency improvement	Vermicompost application rate	Mineral-N treatment	References
Corn	Solid vermicompost	Vermicompost + Bio-organic Fertilizer	Field Trials	Production	+ 6.8%	600 kg/ha (vermicompost); applied on top of optimized N with equivalent N reduction	Optimized N fertilizer 185 kg/ha (urea)	(Li et al., 2011)
Rice	Solid vermicompost	vermicompost	Potted Plant Trials	Production	+ 39%	Soil: earthworm casts = 4:1 (w/w); 7 kg/pot	^15^N-urea 0.74 g/pot (~150 kg N/ha) or 0.44 g/pot (~90 kg N/ha, 60% reduction)	(Huang et al., 2016)
Tomato	Liquid extract (vermiwash/tea)	Foliar Application of Earthworm Extract	Potted Plant Trials	Yield and Quality	Production +120.8%; Vc +46.4%	Foliar VW/VT 0/50/100 mL/L, 5 applications; optimum 100 mL/L	Uniform basal NPK applied to all treatments	(Hamed et al., 2026)
Wheat	Solid vermicompost	Vermicompost + nitrogen fertilizer	Field Trials	Total Nitrogen Uptake	33.21 to 154.33 kg/ha	Vermicompost 0/2.5/5/7.5 t/ha (optimum 5)	Urea N 0/23/46/69 kg/ha (optimum 46)	(Gadisa and Wakgari, 2021)
Soybeans	Solid vermicompost	vermicompost	Potted Plant Trials	Production	+ 18%~60%	40 g/pot (pot diameter 25 cm), ~10 t/ha recommended	No differential mineral-N; 13 treatments managed uniformly; control = no vermicompost	(Dugassa and Worku, 2026)

## 6 Limitations and future directions

Existing research on the nitrogen-promoting effects of vermicompost has systematic shortcomings in terms of methodology, *in situ* mechanisms, product evaluation, and the application of SynComs. There is currently no standardized quantitative separation paradigm for the contributions of substrate and microorganisms to nitrogen fixation; various analytical methods all have inherent errors, and data from different studies lack sufficient cross-study comparability. The mechanisms of nitrogen transformation mediated by the gut and drilosphere are primarily derived from controlled indoor culture systems, and there is a lack of empirical evidence regarding the long-term *in situ* colonization patterns of functional strains under the complex habitats of field conditions. Current quality control for vermicompost covers only physicochemical safety indicators, lacking targeted biological assessment thresholds for nitrogen cycling functional genes and metabolic enzyme activities. A further caveat concerns the attribution of the nitrogen-promoting effect: because most available evidence is correlative (metagenomic enrichment combined with pot experiments), the relative contribution of the microbial module versus the physicochemical substrate (slow-release organic nitrogen, humic-acid buffering, and aggregate-protected nutrient retention) remains unquantified; rigorous partitioning will require sterilization or microbial-inhibition controls that isolate the microbial effect from the substrate effect across field conditions.

Future efforts should focus on systematic technical breakthroughs in quantitative methods, *in situ* field observations, quality control of functional products, the development of nitrogen-promoting microbial communities, and the analysis of tripartite rhizosphere interactions. A further dimension is symbiotic nutrient exchange via arbuscular mycorrhiza, which remains unexplored in the vermicompost context. Mycorrhizal colonization enhances plant nitrogen and phosphorus uptake through dedicated transporters, e.g., the maize mycorrhiza-specific ammonium transporter ZmAMT3;1 and the *Medicago* phosphate transporter MtPT4, while host-derived fatty acids fuel the fungal partner in a reciprocal carbon-for-nitrogen exchange (Javot et al., 2007; Luginbuehl et al., 2017; Hui et al., 2022). These partnerships are stabilized by reciprocal rewards and governed by conserved developmental programs (Kiers et al., 2011; Gutjahr and Parniske, 2013). Integrating mycorrhizal exchange into the substrate–microbial–root framework is a high-priority future direction for symbiotic acquisition. Future research should integrate isotope tracing, multi-omics, and sterile gradient control methods to establish a standardized quantitative decomposition system for the nitrogen-fixing contributions of the substrate and microorganisms. By leveraging long-term fixed-site field platforms, researchers should elucidate the patterns of colonization, succession, and functional decline of functional microbial communities in the field and refine the full-process evaluation system for nitrogen-promoting SynComs, from strain screening to long-term field validation. Furthermore, it is necessary to construct a quality control framework for vermicompost that integrates physicochemical and biological indicators, establishing integrated industrial standards covering raw material composting, finished product testing, and field application. By integrating root-zone multi-omics analysis to elucidate substrate–microbial–crop interaction pathways, we can develop soil- and crop-specific vermicompost application technologies that synergize with fertilizer reduction and efficiency enhancement, thereby supporting efficient and green nitrogen regulation in agricultural fields. Looking forward, the valorization of organic waste streams is expected to diversify beyond biological routes such as vermicomposting, with thermochemical approaches—including gasification—supported by kinetic modeling and machine learning offering complementary pathways.

## 7 Conclusions

This paper establishes a unified analytical framework based on the synergy between the substrate and microbial modules, systematically outlining the full research chain for vermicompost, from microbial community activation mechanisms and the identification of functional bacterial strains to the creation of SynCom and their regulation for industrial-scale application in the field. Earthworms selectively enrich functional microbial communities capable of complete nitrogen cycling through two pathways: gut-associated processes and the microenvironment of the earthworm’s drilosphere. The slow-release nitrogen retention by the substrate and the multi-pathway nitrogen transformation by microorganisms form a temporally coupled synergistic effect, with organic matter providing carbon sources and a microhabitat in the early stages, and functional microbial communities driving nitrogen activation and supply in the later stages. The rational assembly of a nitrogen-promoting SynCom based on indigenous earthworm gut strains holds promise for overcoming the limitations of single-strain adaptation in the field and may represent a key step in the transition from traditional organic fertilizers to precision microbial regulation products. Through targeted regulation of the composting process, physicochemical and biological coupled quality control, and crop-specific application, the synergistic potential to reduce synthetic N fertilizer inputs of vermicompost can be maximized. This comprehensive system provides a conceptual framework and a research roadmap for reducing chemical fertilizer use in agriculture and enhancing soil fertility; key questions regarding field validation, biosafety, and standardization remain to be addressed.

## References

[B1] Abdel Salam M. Roshdy N. M. K. (2022). The influence of different applications of vermicompost tea on the quality of pomegranate fruits. SVU-Int. J. Agric. Sci. 4, 107–118. doi: 10.21608/svuijas.2022.121114.1175

[B2] Adrian P. B. Priya O. V. Chip A. (2016). Nitrogen dynamics of vermicompost use in sustainable agriculture. J. Soil Sci. Environ. Manage. 7, 173–183. doi: 10.5897/JSSEM2016.0587

[B3] Aechra S. Meena R. H. Meena S. C. Mundra S. L. Lakhawat S. S. Mordia A. . (2021). Soil microbial dynamics and enzyme activities as influenced by biofertilizers and split application of vermicompost in rhizosphere of wheat (triticum aestivum. L.). J. Environ. Biol. 42, 1370–1378. doi: 10.22438/jeb/42/5/MRN-1520

[B4] Ahmed N. Al-Mutairi K. A. (2022). Earthworms effect on microbial population and soil fertility as well as their interaction with agriculture practices. Sustainability 14, 7803. doi: 10.3390/su1413780330654563

[B5] Ali S. Liu K. Ahmed W. Jing H. Qaswar M. Kofi Anthonio C. . (2021). Nitrogen mineralization, soil microbial biomass and extracellular enzyme activities regulated by long-term N fertilizer inputs: a comparison study from upland and paddy soils in a red soil region of China. Agronomy 11, 2057. doi: 10.3390/agronomy1110205730654563

[B6] Allouzi M. M. A. Allouzi S. M. A. Keng Z. X. Supramaniam C. V. Singh A. Chong S. (2022). Liquid biofertilizers as a sustainable solution for agriculture. Heliyon 8, e12609. doi: 10.1016/j.heliyon.2022.e12609 36619398 PMC9813699

[B7] Amann R. I. Ludwig W. Schleifer K. H. (1995). Phylogenetic identification and in situ detection of individual microbial cells without cultivation. Microbiol. Rev. 59, 143–169. doi: 10.1128/mr.59.1.143-169.1995 7535888 PMC239358

[B8] Amaya-Gómez C. V. Flórez-Martínez D. H. Cayuela M. L. Tortosa G. (2025). Compost and vermicompost improve symbiotic nitrogen fixation, physiology and yield of the rhizobium-legume symbiosis: a systematic review. Appl. Soil Ecol. 210, 106051. doi: 10.1016/j.apsoil.2025.10605142574925

[B9] Amjadi M. Emami H. Farahani E. Gholoubi A. (2021). Effect of vermicompost and urban waste compost on stability of soil aggregates by high energy moisture characteristic curve. J. Agric. Sci. Technol. 23, 1379–1393.

[B10] Arif M. Pourouchottamane R. Kumar R. Kumar A. Hasanain M. Rai B. . (2026). Integrated nutrient management improves maize productivity, nutrient-use efficiency, soil fertility, and economic returns. J. Agric. Food Res. 27, 102853. doi: 10.1016/j.jafr.2026.10285342574925

[B11] Askari A. Khanmirzaei A. Rezaei S. (2020). Vermicompost enrichment using organic wastes: nitrogen content and mineralization. Int. J. Recycl. Org. Waste. Agric. 9, 151–160. doi: 10.30486/ijrowa.2020.1885015.1001

[B12] Atiyeh R. Lee S. Edwards C. Arancon N. Metzger J. (2002). The influence of humic acids derived from earthworm-processed organic wastes on plant growth. Bioresour. Technol. 84, 7–14. doi: 10.1016/S0960-8524(02)00017-2 12137272

[B13] Bary A. Cogger C. Sullivan D. (2002). What Does Compost Analysis Tell You About Your Compost? Available online at: https://puyallup.wsu.edu/soils/compost/compost-analysis/ (Accessed July 18, 2026).

[B14] Bernai M. P. Paredes C. Sánchez-Monedero M. A. Cegarra J. (1998). Maturity and stability parameters of composts prepared with a wide range of organic wastes. Bioresour. Technol. 63, 91–99. doi: 10.1016/S0960-8524(97)00084-9

[B15] Bouguyon E. Brun F. Meynard D. Kubeš M. Pervent M. Leran S. . (2015). Multiple mechanisms of nitrate sensing by arabidopsis nitrate transceptor NRT1.1. Nat. Plants 1, 15015. doi: 10.1038/nplants.2015.15 27246882

[B16] Canning A. D. (2026). Vermicompost alters soil microbial communities and decomposition but increases nitrate leaching in tropical sugarcane. Ecol. Evol. 16, e72925. doi: 10.1002/ece3.72925 41583890 PMC12828582

[B17] Chen C. Whalen J. K. (2016). Earthworm interactions with denitrifying bacteria are scale-dependent: evidence from physiological to riparian ecosystem scales. Basic. Appl. Ecol. 17, 1–10. doi: 10.1016/j.baae.2015.12.00742574925

[B18] Chiranjeeb K. Prasad S. S. (2020). Preparation of quality enriched vermicompost and its analysis. Int. J. Curr. Microbiol. Appl. Sci. 9, 3556–3571. doi: 10.20546/ijcmas.2020.907.416

[B19] Choi S. Hwang H.-S. Lee H.-S. (2026). Designing field-ready biocontrol: discovery–syncom–validation. Plant Biotechnol. Rep. 20, 1–10. doi: 10.1007/s11816-025-01029-030311153

[B20] Compant S. Samad A. Faist H. Sessitsch A. (2019). A review on the plant microbiome: ecology, functions, and emerging trends in microbial application. J. Adv. Res. 19, 29–37. doi: 10.1016/j.jare.2019.03.004 31341667 PMC6630030

[B21] Coq S. Barthès B. G. Oliver R. Rabary B. Blanchart E. (2007). Earthworm activity affects soil aggregation and organic matter dynamics according to the quality and localization of crop residues—an experimental study (Madagascar). Soil Biol. Biochem. 39, 2119–2128. doi: 10.1016/j.soilbio.2007.03.01942574925

[B22] Daims H. Lebedeva E. V. Pjevac P. Han P. Herbold C. Albertsen M. . (2015). Complete nitrification by nitrospira bacteria. Nature 528, 504–509. doi: 10.1038/nature16461 26610024 PMC5152751

[B23] De Souza R. S. C. Armanhi J. S. L. Arruda P. (2020). From microbiome to traits: designing synthetic microbial communities for improved crop resiliency. Front. Plant Sci. 11, 1179. doi: 10.3389/fpls.2020.01179 32983187 PMC7484511

[B24] Devliegher W. Verstraete W. (1995). Lumbricus terrestris in a soil core experiment: Nutrient-enrichment processes (NEP) and gut-associated processes (GAP) and their effect on microbial biomass and microbial activity. Soil Biol. Biochem. 27, 1573–1580. doi: 10.1016/0038-0717(95)00090-2

[B25] Domínguez J. Lazcano C. Gómez-Brandón M. (2010). Influencia del vermicompost en el crecimiento de las plantas. Aportes para la elaboración de un concepto objetivo. Acta Zool. Mex. Ns. 26, 359–371. doi: 10.21829/azm.2010.262900

[B26] Dong C.-J. Liu X.-Y. Xie L.-L. Wang L.-L. Shang Q.-M. (2020). Salicylic acid regulates adventitious root formation via competitive inhibition of the auxin conjugation enzyme CsGH3.5 in cucumber hypocotyls. Planta 252, 75. doi: 10.1007/s00425-020-03467-2 33026530

[B27] Drake H. L. Horn M. A. (2007). As the worm turns: the earthworm gut as a transient habitat for soil microbial biomes. Annu. Rev. Microbiol. 61, 169–189. doi: 10.1146/annurev.micro.61.080706.093139 17506687

[B28] Du H.-Q. Sun H. Zhou J. Zhong H. Li X.-L. Peng F. . (2026). Microbial mechanisms enhancing soil phosphorus bioavailability through vermicompost application for sustainable agriculture. Eng. Agric. 13, 26672. doi: 10.15302/J-FASE-2026672

[B29] Ducasse V. Capowiez Y. Peigné J. (2022). Vermicomposting of municipal solid waste as a possible lever for the development of sustainable agriculture. A review. Agron. Sustain. Dev. 42, 89–105. doi: 10.1007/s13593-022-00819-y30311153

[B30] Dugassa M. Worku W. (2026). Comparison of effects of different vermicompost types on growth, nodule formation, and yield response of soybean. Sci. Rep. 16, 16093. doi: 10.1038/s41598-026-47574-8 41963475 PMC13199505

[B31] Ebrahimi E. Ghorbani R. Von Fragstein Und Niemsdorff P. (2020). Effects of vermicompost placement on nutrient use efficiency and yield of tomato (*Lycopersicum esculentum*). Biol. Agric. Hortic. 36, 44–52. doi: 10.1080/01448765.2019.167122037339054

[B32] European Commission (2010). Soil Biodiversity: Functions, Threats and Tools for Policy Makers. Final Report (Brussels: European Commission). doi: 10.2779/14571

[B33] Farhangi-Abriz S. Alaee T. Tavasolee A. (2019). Salicylic acid but not jasmonic acid improved canola root response to salinity stress. Rhizosphere 9, 69–71. doi: 10.1016/j.rhisph.2018.11.00942574925

[B34] Fikru T. K. Fikreyohannes G. (2018). Response of garlic (allium sativum L.) to vermicompost and mineral N fertilizer application at haramaya, eastern Ethiopia. Afr. J. Agric. Res. 13, 27–35. doi: 10.5897/AJAR2017.12708

[B35] Food and Agriculture Organization (2025). The State of Food and Agriculture 2025 (Rome: FAO). doi: 10.4060/cd7067en

[B36] Gadisa N. Wakgari T. (2021). Nitrogen uptake and use efficiency of wheat (*triticum aestivum* L.) varieties as influenced by combined application of vermicompost and nitrogen fertilizer rate. Am. J. Biosci. Bioeng. 9, 169–178. doi: 10.11648/j.bio.20210906.14

[B37] Garcia J. Moravek M. Fish T. Thannhauser T. Fei Z. Sparks J. P. . (2024). Rhizosphere microbiomes derived from vermicompost alter gene expression and regulatory pathways in tomato (solanum lycopersicum, L.). Sci. Rep. 14, 21362. doi: 10.1038/s41598-024-71792-7 39266588 PMC11393070

[B38] Garg P. Gupta A. Satya S. (2006). Vermicomposting of different types of waste using eisenia foetida: a comparative study. Bioresour. Technol. 97, 391–395. doi: 10.1016/j.biortech.2005.03.009 16168639

[B39] Ge Y. Yang A. Dong Y. Wang S. Xu R. Zhou D. (2025). Efficient degradation mechanism of fomesafen by earthworms and gut degrading bacteria synthetic community. Pestic. Biochem. Physiol. 214, 106607. doi: 10.1016/j.pestbp.2025.106607 40915799

[B40] Geelani S. B. Saraf A. Das P. (2026). A review on novel fertilizer technologies as climate-smart solutions for mitigating greenhouse gas emissions from croplands. Clim. Smart. Agric. 3, 100123. doi: 10.1016/j.csag.2026.10012342574925

[B41] Giehl R. F. H. Von Wiren N. (2014). Root nutrient foraging. Plant Physiol. 166, 509–517. doi: 10.1104/pp.114.245225 25082891 PMC4213083

[B42] Großkopf T. Soyer O. S. (2014). Synthetic microbial communities. Curr. Opin. Microbiol. 18, 72–77. doi: 10.1016/j.mib.2014.02.002 24632350 PMC4005913

[B43] Gruber B. D. Giehl R. F. H. Friedel S. Von Wirén N. (2013). Plasticity of the arabidopsis root system under nutrient deficiencies. Plant Physiol. 163, 161–179. doi: 10.1104/pp.113.218453 23852440 PMC3762638

[B44] Gutjahr C. Parniske M. (2013). Cell and developmental biology of arbuscular mycorrhiza symbiosis. Annu. Rev. Cell Dev. Biol. 29, 593–617. doi: 10.1146/annurev-cellbio-101512-122413 24099088

[B45] Habtamu M. Elias E. Soromessa T. Argaw M. (2024). Effects of integrated application of vermicompost and inorganic fertilizers on selected soil characteristics and productivity of wheat (*Triticum aestivum* L.) and faba bean (*Vicia faba* L.) in dire and legedadi watersheds of Ethiopia. Appl. Environ. Soil Sci. 2024, 3163750. doi: 10.1155/2024/316375040201959

[B46] Hamed M. H. Al-Obayki O. I. Al-Nasser A. M. Al-Oudah S. A. Al-Eid S. M. (2026). Boosting organic tomato yield/quality: foliar vermiwash and vermicompost tea in soilless and soil-based systems greenhouse. Org. Agric. 16, 5–16. doi: 10.1007/s13165-025-00537-130311153

[B47] Hepşen Türkay F.Ş. (2025). Quality assessment of farmer-led vermicompost production in semi-arid agroecosystems: compliance with global standards. Sustainability 18, 298–320. doi: 10.3390/su1801029830654563

[B48] Hoque T. S. Hasan A. K. Hasan M. A. Nahar N. Dey D. K. Mia S. . (2022). Nutrient release from vermicompost under anaerobic conditions in two contrasting soils of Bangladesh and its effect on wetland rice crop. Agriculture 12, 376–392. doi: 10.3390/agriculture1203037630654563

[B49] Horn M. A. Ihssen J. Matthies C. Schramm A. Acker G. Drake H. L. (2005). Dechloromonas denitrificans sp. nov. flavobacterium denitrificans sp. nov. paenibacillus anaericanus sp. nov. and paenibacillus terrae strain MH72, N2O-producing bacteria isolated from the gut of the earthworm aporrectodea caliginosa. Int. J. Syst. Evol. Microbiol. 55, 1255–1265. doi: 10.1099/ijs.0.63484-0 15879265

[B50] Huang K. Liu W. Xia H. (2023). “ Metagenomic analysis revealing the dual microbial community features in three common vermicomposts,” in Fate of Biological Contaminants During Recycling of Organic Wastes (Amsterdam: Elsevier), 157–176. doi: 10.1016/B978-0-323-95998-8.00003-0

[B51] Huang M. Zhou X. Xie X. Zhao C. Chen J. Cao F. . (2016). Rice yield and the fate of fertilizer nitrogen as affected by addition of earthworm casts collected from oilseed rape fields: a pot experiment. PloS One 11, e0167152. doi: 10.1371/journal.pone.0167152 27880837 PMC5120869

[B52] Hui J. An X. Li Z. Neuhäuser B. Ludewig U. Wu X. . (2022). The mycorrhiza-specific ammonium transporter ZmAMT3;1 mediates mycorrhiza-dependent nitrogen uptake in maize roots. Plant Cell 34, 4066–4087. doi: 10.1093/plcell/koac225 35880836 PMC9516061

[B53] Hussain N. Singh A. Saha S. Venkata Satish Kumar M. Bhattacharyya P. Bhattacharya S. S. (2016). Excellent N-fixing and P-solubilizing traits in earthworm gut-isolated bacteria: a vermicompost based assessment with vegetable market waste and rice straw feed mixtures. Bioresour. Technol. 222, 165–174. doi: 10.1016/j.biortech.2016.09.115 27718399

[B54] International Organization For Standardization (2012). Soil Quality — Biological Methods — Determination of Nitrogen Mineralization and Nitrification in Soils and the Influence of Chemicals on These Processes. Available online at: https://www.iso.org/standard/56033.html (Accessed July 18, 2026).

[B55] Jat O. P. Das P. (2026). Isolation and identification of bacillus subtilis and pseudomonas fluorescens bacteria from cotton plant (gossypium hirsutum) rhizospheric soil and potential use as food waste decomposer. Sustain. 8, 100394. doi: 10.1016/j.nxsust.2026.10039442574925

[B56] Javanmardi J. Dos Santos M. L. Manter D. K. Vivanco J. M. (2024). Worm compost tea’s plant growth-promoting power: is it the microorganisms or chemical properties? J. Plant Nutr. 47, 583–594. doi: 10.1080/01904167.2023.228013337339054

[B57] Javot H. Penmetsa R. V. Terzaghi N. Cook D. R. Harrison M. J. (2007). A *Medicago truncatula* phosphate transporter indispensable for the arbuscular mycorrhizal symbiosis. Proc. Natl. Acad. Sci. 104, 1720–1725. doi: 10.1073/pnas.0608136104 17242358 PMC1785290

[B58] Jeevapriya J. Kanimozhi A. (2024). A review on bacterial enriched vermicompost and its applications. Int. J. Zool. Appl. Biosci. 9, 51–54. doi: 10.55126/ijzab.2024.v09.i03.009

[B59] Jiang M. Dong C. Bian W. Zhang W. Wang Y. (2024). Effects of different fertilization practices on maize yield, soil nutrients, soil moisture, and water use efficiency in northern China based on a meta-analysis. Sci. Rep. 14, 6480. doi: 10.1038/s41598-024-57031-z 38499586 PMC10948899

[B60] Jiang S. Li H. Zhang L. Mu W. Zhang Y. Chen T. . (2025). Generic Diagramming Platform (GDP): a comprehensive database of high-quality biomedical graphics. Nucleic Acids Res. 53, D1670–D1676. doi: 10.1093/nar/gkae973 39470721 PMC11701665

[B61] Jiménez J. J. Decaëns T. Lavelle P. Thomas R. J. (2001). “ Native earthworm communities, soil processes and sustainability of Colombian savannas,” in Food and Agriculture Organization of the United Nations (Fao) ( Centro Internacional de Agricultura Tropical, Cali, CO), 1–14. Available online at: https://www.fao.org/fileadmin/templates/agphome/scpi/SCPI_Compendium/Native_earthworm_communities.pdf (Accessed July 18, 2026).

[B62] Jin B.-J. Liu X.-P. Roux X. L. Bi Q.-F. Li K.-J. Wu C.-Y. . (2022). Biochar addition regulates soil and earthworm gut microbiome and multifunctionality. Soil Biol. Biochem. 173, 108810. doi: 10.1016/j.soilbio.2022.10881042574925

[B63] Joshi R. Singh J. Vig A. P. (2015). Vermicompost as an effective organic fertilizer and biocontrol agent: effect on growth, yield and quality of plants. Rev. Environ. Sci. Bio/Technol. 14, 137–159. doi: 10.1007/s11157-014-9347-130311153

[B64] Jouquet P. Bottinelli N. Podwojewski P. Hallaire V. Tran Duc T. (2008). Chemical and physical properties of earthworm casts as compared to bulk soil under a range of different land-use systems in Vietnam. Geoderma 146, 231–238. doi: 10.1016/j.geoderma.2008.05.03042574925

[B65] Karine S. Elizaveta G. Valeriia K. Andrey V. Andrey G. (2024). Comparative intestine microbiome dynamics of the earthworm eisenia fetida cultivated in sewage sludge and peat. Bioresour. Technol. Rep. 27, 101948. doi: 10.1016/j.biteb.2024.10194842574925

[B66] Kerketta V. Panda A. K. Kerketta A. De Mandal S. Bisht S. S. (2025). Molecular characterization of phosphate solubilizing bacteria klebsiella variicola PSEG-1 associated with aporrectodea rosea gastrointestinal tract. Bacteria 4, 5–14. doi: 10.3390/bacteria401000530654563

[B67] Kiers E. T. Duhamel M. Beesetty Y. Mensah J. A. Franken O. Verbruggen E. . (2011). Reciprocal rewards stabilize cooperation in the mycorrhizal symbiosis. Science 333, 880–882. doi: 10.1126/science.1208473 21836016

[B68] Kim B.-C. R. Kleinsteuber S. Lawson C. E. (2025). Carbon-efficient waste upcycling: combining syngas fermentation and chain elongation with synthetic consortia. Curr. Opin. Biotechnol. 94, 103321. doi: 10.1016/j.copbio.2025.103321 40532266

[B69] Kong Y. Zhang J. Zhang X. Gao X. Yin J. Wang G. . (2024). Applicability and limitation of compost maturity evaluation indicators: a review. Chem. Eng. J. 489, 151386. doi: 10.1016/j.cej.2024.15138642574925

[B70] Kováčik P. Šimanský V. Kmeťová M. Týr Š. Ledwożyw-Smoleń I. (2026). Effect of dose and date of application of vermicompost and its combination with N-fertilizer on maize grain yield. Agronomy 16, 118–130. doi: 10.3390/agronomy1601011830654563

[B71] Kumar D. S. Kumar P. S. Rajendran N. M. Anbuganapathi G. (2013). Compost maturity assessment using physicochemical, solid-state spectroscopy, and plant bioassay analysis. J. Agric. Food. Chem. 61, 11326–11331. doi: 10.1021/jf4034943 24191667

[B72] Kumar Sarker A. Kuar K. D. Kuriakose E. Morton C. O. Stack C. M. Moffitt M. C. (2026). Beyond the single isolate: leveraging plant-associated microbial communities for crop resilience. Microorganisms 14, 456–474. doi: 10.3390/microorganisms14020456 41753742 PMC12942860

[B73] Lastiri-Hernández M. A. Álvarez-Bernal D. Barajas E. C. Magallón R. F. García J. T. S. Cárdenas G. C. (2023). Effects of sesuvium verrucosum raf. Compost and vermicompost on the growth and production parameters of the solanum lycopersicum L. crop. Bragantia 82, e20220162. doi: 10.1590/1678-4499.2022016241099703

[B74] Leicht K. M. (2024). Development of a comprehensive nitrogen budget to increase nitrogen use efficiency and reduce nitrogen losses in semi-arid southern alberta. Univ. Alberta. doi: 10.7939/R3-BTJP-5D77

[B75] León-Anzueto E. Abud-Archila M. Dendooven L. Ventura-Canseco L. M. C. Gutiérrez-Miceli F. A. (2011). Effect of vermicompost, worm-bed leachate and arbuscular mycorrizic fungi on lemongrass (cymbopogon citratus stapf.) growth and composition of its essential oils. Electron. J. Biotechnol. 14, 1–11. doi: 10.2225/vol14-issue6-fulltext-9

[B76] Lewis W. H. Tahon G. Geesink P. Sousa D. Z. Ettema T. J. G. (2021). Innovations to culturing the uncultured microbial majority. Nat. Rev. Microbiol. 19, 225–240. doi: 10.1038/s41579-020-00458-8 33093661

[B77] Li H. Xiang D. Li X. Wang C. Dong M. (2011). The effects of earthworm castings and bio-organic fertilizer on soil nutrients and summer corn yields. Soil Bull. 42, 1179–1183. doi: 10.19336/j.cnki.trtb.2011.05.029

[B78] Li F. Xu M.-B. Pan L.-H. Li J. Lan C.-B. Li Z. . (2025). Ammonifying and phosphorus-solubilizing function of aliikangiella maris sp. nov. isolated from phaeocystis globosa bloom and algal–bacterial interactions. Front. Microbiol. 16, 1516993. doi: 10.3389/fmicb.2025.1516993 39996082 PMC11849500

[B79] Li J. Zhang Y. Huang K. Xia H. (2020). Metagenomic analysis of the composition of microbial populations and antibiotic-resistance genes in earthworm castings. Chin. Environ. Sci. 40, 5375–5382. doi: 10.19674/j.cnki.issn1000-6923.2020.0594

[B80] Liu M. Cao J. Wang C. Wang B. Xue R. (2024). Vermicompost enhances the salt tolerance of maize by reshaping the rhizosphere microenvironment. Appl. Soil Ecol. 203, 105633. doi: 10.1016/j.apsoil.2024.10563342574925

[B81] Liu M. Wang C. Liu X. Lu Y. Wang Y. (2020). Saline-alkali soil applied with vermicompost and humic acid fertilizer improved macroaggregate microstructure to enhance salt leaching and inhibit nitrogen losses. Appl. Soil Ecol. 156, 103705. doi: 10.1016/j.apsoil.2020.10370542574925

[B82] Lu Y. Qin T. Chen W. Cai J. Xu Y. Li Y. . (2024). Effects of earthworm castings application on soil organic carbon pools and their stability in coastal saline-alkali lands. Soils 56, 817–825. doi: 10.13758/j.cnki.tr.2024.04.015

[B83] Luginbuehl L. H. Menard G. N. Kurup S. Van Erp H. Radhakrishnan G. V. Breakspear A. . (2017). Fatty acids in arbuscular mycorrhizal fungi are synthesized by the host plant. Science 356, 1175–1178. doi: 10.1126/science.aan0081 28596311

[B84] Ma Q. Zhang B. Cui J. (2026). The effect of indigenous cultivable microorganism inoculation on soil microecology during restoration of obstructed soils. Microorganisms 14, 784–802. doi: 10.3390/microorganisms14040784 42075181 PMC13118302

[B85] Mahmood A. Turgay O. C. Farooq M. Hayat R. (2016). Seed biopriming with plant growth promoting rhizobacteria: a review. FEMS Microbiol. Ecol. 92, fiw112. doi: 10.1093/femsec/fiw112 27222220

[B86] Maji D. Misra P. Singh S. Kalra A. (2017). Humic acid rich vermicompost promotes plant growth by improving microbial community structure of soil as well as root nodulation and mycorrhizal colonization in the roots of pisum sativum. Appl. Soil Ecol. 110, 97–108. doi: 10.1016/j.apsoil.2016.10.00842574925

[B87] Manivannan N. Daniel T. (2009). Use of vermicompost as carrier material for microbial inoculants for enhanced crop production. J. Pure. Appl. Microbiol. 3, 255–260.

[B88] Mapile M. R. F. Obusan M. C. M. (2020). Mineralization, biodegradation, and antagonistic activities of gut-associated bacteria and fungi of African nightcrawler, eudrilus eugeniae (kinberg 1867). Sci. Diliman. 32, 1–26.

[B89] Meena R. Ram H. Saxena A. Kumar R. (2024). Natural Farming: Basics and Application (Karnal: ICAR-NDRI).

[B90] Miao Y. Sun X. Wang W. Shen Q. Zhang R. (2026). Dynamical model-guided SynCom design for sustainable agriculture. Trends Microbiol. 34, 828–839. doi: 10.1016/j.tim.2026.04.008 42120207

[B91] Ministry Of Agriculture And Rural Affairs Of The People’s Republic Of China (2012). Bio-organic fertilizer. (Beijing: Standards Press of China).

[B92] Ministry Of Agriculture And Rural Affairs Of The People’s Republic Of China (2015). Compound microbial fertilizer. (Beijing: Standards Press of China).

[B93] Ministry Of Agriculture And Rural Affairs Of The People’s Republic Of China (2021). Organic fertilizer. (Beijing: Standards Press of China).

[B94] Mirleau P. Jouni F. Chappat J. Mazzia C. Sanchez-Hernandez J. C. Capowiez Y. . (2024). Effect of an organophosphorus insecticide, soil texture and earthworm species on the turnover of soil, gut and cast microbiota during the earthworm’s gut transit. Soil Biol. Biochem. 190, 109293. doi: 10.1016/j.soilbio.2023.10929342574925

[B95] Mohd Zuki M. M. Md. Jaafar N. Sakimin S. Z. Yusop M. K. (2020). N-(n-butyl) thiophosphoric triamide (NBPT)-coated urea (NCU) improved maize growth and nitrogen use efficiency (NUE) in highly weathered tropical soil. Sustainability 12, 8780. doi: 10.3390/su1221878030654563

[B96] Molla H. Gashaw M. Wassie H. (2019). Nutrient release pattern from leptic cambisols as influenced by vermicompost and inorganic fertilizer applications. J. Soil Sci. Environ. Manage. 10, 12–20. doi: 10.5897/JSSEM2018.0717

[B97] Moreira M. D. F. Santos P. R. D. (2012). Tratamento orgânico de resíduos de rúmen bovino. Undergrad. Thesis. Universidade. Estadual. Paul. “Júlio. Mesquita. Filho,”, 1–63.

[B98] Mubaraq A. Sembiring M. Widiastuti E. Fachrial E. Utomo B. Turjaman M. . (2024). Diversity and distribution of nitrifying bacteria play an important role in the nitrogen cycle in mangrove sediments. Glob. J. Environ. Sci. Manage. 10, 1–18. doi: 10.22034/gjesm.2024.04.39

[B99] Munnoli P. M. Bhosle S. (2011). Water-holding capacity of earthworms’ vermicompost made of sugar industry waste (press mud) in mono- and polyculture vermireactors. Environmentalist 31, 394–400. doi: 10.1007/s10669-011-9353-630311153

[B100] Nath S. Qureshi A. Das S. (2023). Role of bulking agents, process optimization, and different earthworm species in the vermiremediation process of industrial wastes: a review. Not. Sci. Biol. 15, 11490. doi: 10.55779/nsb15211490

[B101] Nechayeva A. Boyarshin K. Bespalova O. Klyueva V. Makanina O. Batlutskaya I. (2021). Analysis of 16S rRNA gene variability in soil nitrifying bacteria of the genus *Nitrosomonas*. Bio Web Conf. 30, 5007. doi: 10.1051/bioconf/2021300500724184215

[B102] Niu T. Wang H. Fu W. Hao Z. Wu S. Zhang X. . (2026). Multifunctional composite microbial inoculants in sustainable agriculture: design, carriers and field application. Soil Ecol. Lett. 8, 260421. doi: 10.1007/s42832-026-0421-830311153

[B103] Nonthapa A. Iwai C. B. Chankaew S. Falab S. (2024). Dual-purpose vermicompost for the growth promotion and suppression of damping-off disease on potted vegetable soybean. Plants 13, 1607. doi: 10.3390/plants13121607 38931039 PMC11207718

[B104] Ojeda-Morales M. E. Domínguez-Domínguez M. Hernández-Rivera M. A. Zavala-Cruz J. (2015). Biosurfactant production by strains of azospirillum isolated from petroleum-contaminated sites. Water Air. Soil pollut. 226, 401–415. doi: 10.1007/s11270-015-2659-030311153

[B105] Olaria M. Nebot J. F. Molina H. Troncho P. Lapeña L. Llorens E. (2016). Effect of different substrates for organic agriculture in seedling development of traditional species of solanaceae. Span. J. Agric. Res. 14, e0801. doi: 10.5424/sjar/2016141-801342627795

[B106] Oldroyd G. E. D. Leyser O. (2020). A plant’s diet, surviving in a variable nutrient environment. Science 368, eaba0196. doi: 10.1126/science.aba0196 32241923

[B107] Omokaro G. O. (2024). Effects of earthworms on microbial communities and their castings as soil amendments: a review. doi: 10.2139/ssrn.4877681

[B108] Oyserman B. O. Medema M. H. Raaijmakers J. M. (2018). Road MAPs to engineer host microbiomes. Curr. Opin. Microbiol. 43, 46–54. doi: 10.1016/j.mib.2017.11.023 29207308

[B109] Pereira M. G. Arruda M. A. Z. (2003). Vermicompost as a natural adsorbent material: characterization and potentialities for cadmium adsorption. J. Braz. Chem. Soc 14, 39–47. doi: 10.1590/S0103-5053200300010000741099703

[B110] Pishgar R. Dominic J. A. Tay J. H. Chu A. (2021). Changes in BNR microbial community in response to different selection pressure. Nitrogen 2, 474–490. doi: 10.3390/nitrogen204003230654563

[B111] Prosser J. I. Nicol G. W. (2012). Archaeal and bacterial ammonia-oxidisers in soil: the quest for niche specialisation and differentiation. Trends Microbiol. 20, 523–531. doi: 10.1016/j.tim.2012.08.001 22959489

[B112] Qi Y. (2012). Vermiculture technology: earthworms, organic wastes, and environmental management. Int. J. Environ. Stud. 69, 173–174. doi: 10.1080/00207233.2012.62432737339054

[B113] Quirós P. Sala-Comorera L. Gómez-Gómez C. Ramos-Barbero M. D. Rodríguez-Rubio L. Vique G. . (2023). Identification of a virulent phage infecting species of *Nitrosomonas*. ISME. J. 17, 645–648. doi: 10.1038/s41396-023-01380-6 36759553 PMC10119301

[B114] Rabara S. Vishwakarma N. P. Patel S. (2023). Isolation and biochemical identification of N2 fixing bacteria (azospirillium sp.) from saurashtra region. Curr. Agric. Res. J. 11, 277–286. doi: 10.12944/CARJ.11.1.24

[B115] Rakkini V. M. Priya F. J. David R. D. Malakondaiah S. Menakha M. Sylvia S. M. . (2025). A review of the role of vermicompost in enhancing crop productivity and soil health. Acad. Soc 12, 21–34. doi: 10.6025/aas/2025/12/1/21-34

[B116] Robe Elema W. (2021). Preparation and characterization of vermicompost made from different sources of materials. Open J. Plant Sci. 6 (1), 42–48. doi: 10.17352/ojps.000031

[B117] Rocha I. Ma Y. Souza-Alonso P. Vosátka M. Freitas H. Oliveira R. S. (2019). Seed coating: a tool for delivering beneficial microbes to agricultural crops. Front. Plant Sci. 10, 1357. doi: 10.3389/fpls.2019.01357 31781135 PMC6852281

[B118] Sakpirom J. Nunkaew T. Khan E. Kantachote D. (2021). Optimization of carriers and packaging for effective biofertilizers to enhance oryza sativa L. growth in paddy soil. Rhizosphere 19, 100383. doi: 10.1016/j.rhisph.2021.10038342574925

[B119] Sande T. J. Tindwa H. J. Alovisi A. M. T. Shitindi M. J. Semoka J. M. (2024). Enhancing sustainable crop production through integrated nutrient management: a focus on vermicompost, bio-enriched rock phosphate, and inorganic fertilisers – a systematic review. Front. Agron. 6, 1422876. doi: 10.3389/fagro.2024.1422876

[B120] Sarkar R. Mukherjee S. Pradhan B. Chatterjee G. Goswami R. Ali M. N. . (2023). Molecular characterization of vermicompost-derived IAA-releasing bacterial isolates and assessment of their impact on the root improvement of banana during primary hardening. World J. Microbiol. Biotechnol. 39, 351–363. doi: 10.1007/s11274-023-03809-8 37864056

[B121] Shayanthan A. Ordoñez P. A. C. Oresnik I. J. (2022). The role of synthetic microbial communities (SynCom) in sustainable agriculture. Front. Agron. 4, 896307. doi: 10.3389/fagro.2022.896307

[B122] Sidorova N. A. Chechkova N. A. (2021). Results of primary screening of proteolytically active microorganisms that are promising for the transformation of organic pollution. IOP. Conf. Ser. Earth Environ. Sci. 689, 12041. doi: 10.1088/1755-1315/689/1/012041

[B123] Singh A. Singh D. P. Tiwari R. Kumar K. Singh R. V. Singh S. . (2015). Taxonomic and functional annotation of gut bacterial communities of eisenia foetida and perionyx excavatus. Microbiol. Res. 175, 48–56. doi: 10.1016/j.micres.2015.03.003 25813857

[B124] Sinha R. Herat S. Valani D. Chauhan K. (2009). Earthworms vermicompost: a powerful crop nutrient over the conventional compost & protective soil conditioner against the destructive chemical fertilizers for food safety and security. Am.-Eurasian. J. Agric. Environ. Sci. 5, 1–55.

[B125] Soltan H. A. H. Dakhly O. F. Mahmoud M. A. Fayz Y. F. M. (2022). Microbiological and genetical identification of some vermicompost beneficial associated bacteria. SVU-Int. J. Agric. Sci. 4, 21–36. doi: 10.21608/svuijas.2021.106875.1154

[B126] Spieck E. Bock E. (2005). “ Nitrifying bacteria,” in Boston (Boston: Springer, Boston, MA). doi: 10.1007/0-387-28021-9_17

[B127] Standards Australia (2012). Composts, soil conditioners and mulches. (Sydney).

[B128] Taheri S. (2018). Macro and micro-evolutionary processes within a complex of species, case study of the tropical invasive earthworm: pontoscolex corethrurus (Creteil: Université Paris-Est). doi: 10.70675/ebda1d02z54fdz412aza468z2060a0db4da4

[B129] Taleski M. Imin N. Djordjevic M. A. (2018). CEP peptide hormones: key players in orchestrating nitrogen-demand signalling, root nodulation, and lateral root development. J. Exp. Bot. 69, 1829–1836. doi: 10.1093/jxb/ery037 29579226

[B130] Tan J. Huang H. Tang W. Zhu Wang D. (2021). The effects of earthworm castings as organic fertilizer on soil microbial communities. Jiangsu. Agric. Sci. 49, 228–233. doi: 10.15889/j.issn.1002-1302.2021.20.037

[B131] Tavali I. E. Uz I. (2025). Comparison of heat-treated and untreated vermicompost as a peat substitute in seedling growing media for lettuce seedling growth and microbial properties. BMC Plant Biol. 25, 1240. doi: 10.1186/s12870-025-07256-5 41029221 PMC12482194

[B132] Thakuria D. Schmidt O. Finan D. Egan D. Doohan F. M. (2010). Gut wall bacteria of earthworms: a natural selection process. ISME. J. 4, 357–366. doi: 10.1038/ismej.2009.124 19924156

[B133] Tian X. Han B. Liang J. Yang F. Zhang K. (2021). Tracking antibiotic resistance genes (ARGs) during earthworm conversion of cow dung in northern China. Ecotoxicol. Environ. Saf. 222, 112538. doi: 10.1016/j.ecoenv.2021.112538 34325199

[B134] Trivedi P. Mattupalli C. Eversole K. Leach J. E. (2021). Enabling sustainable agriculture through understanding and enhancement of microbiomes. New Phytol. 230, 2129–2147. doi: 10.1111/nph.17319 33657660

[B135] Van Groenigen J. W. Lubbers I. M. Vos H. M. J. Brown G. G. De Deyn G. B. Van Groenigen K. J. (2014). Earthworms increase plant production: a meta-analysis. Sci. Rep. 4, 6365. doi: 10.1038/srep06365 25219785 PMC5376159

[B136] Van Kessel M. A. H. J. Speth D. R. Albertsen M. Nielsen P. H. Op Den Camp H. J. M. Kartal B. . (2015). Complete nitrification by a single microorganism. Nature 528, 555–559. doi: 10.1038/nature16459 26610025 PMC4878690

[B137] Velásquez-Chávez T. E. Sáenz-Mata J. Quezada-Rivera J. J. Palacio-Rodríguez R. Muro-Pérez G. Servín-Prieto A. J. . (2025). Bacterial and physicochemical dynamics during the vermicomposting of bovine manure: a comparative analysis of the eisenia fetida gut and compost matrix. Microbiol. Res. 16, 177–198. doi: 10.3390/microbiolres1608017730654563

[B138] Vidal A. Watteau F. Remusat L. Mueller C. W. Nguyen Tu T.-T. Buegger F. . (2019). Earthworm cast formation and development: a shift from plant litter to mineral associated organic matter. Front. Environ. Sci. 7. doi: 10.3389/fenvs.2019.00055

[B139] Vos H. M. J. Koopmans G. F. Beezemer L. De Goede R. G. M. Hiemstra T. Van Groenigen J. W. (2019). Large variations in readily-available phosphorus in casts of eight earthworm species are linked to cast properties. Soil Biol. Biochem. 138, 107583. doi: 10.1016/j.soilbio.2019.10758342574925

[B140] Vuković A. Velki M. Ečimović S. Vuković R. Štolfa Čamagajevac I. Lončarić Z. (2021). Vermicomposting—Facts, benefits and knowledge gaps. Agronomy 11, 1952. doi: 10.3390/agronomy1110195230654563

[B141] Walpola B. Wanniarachchi S. (2009). Microbial respiration and nitrogen mineralization in soil amended with different proportions of vermicompost and coir dust. Bangladesh. J. Agric. Res. 34, 537–543. doi: 10.3329/bjar.v34i4.583040208441

[B142] Wang Z. Wang S. Liu Y. Feng K. Deng Y. (2018). Application of metagenomic technology in the molecular detection of functional microorganisms in the nitrogen cycle. Bio/Technol. Bull. 34, 1–14. doi: 10.13560/j.cnki.biotech.bull.1985.2018-0024

[B143] Wüst P. K. Horn M. A. Drake H. L. (2009). In situ hydrogen and nitrous oxide as indicators of concomitant fermentation and denitrification in the alimentary canal of the earthworm Lumbricus terrestris. Appl. Environ. Microbiol. 75, 1852–1859. doi: 10.1128/AEM.02745-08 19201982 PMC2663201

[B144] Wüst P. K. Horn M. A. Drake H. L. (2011). Clostridiaceae and Enterobacteriaceae as active fermenters in earthworm gut content. ISME. J. 5, 92–106. doi: 10.1038/ismej.2010.99 20613788 PMC3105676

[B145] Xue R. Wang C. Liu X. Liu M. (2022). Earthworm regulation of nitrogen pools and dynamics and marker genes of nitrogen cycling: a meta-analysis. Pedosphere 32, 131–139. doi: 10.1016/S1002-0160(21)60063-2

[B146] Yang Z. Luo Y. Chen H. Zhang Y. Wu S. Yang M. . (2024b). Vermicompost addition improved soil aggregate stability, enzyme activity, and soil available nutrients. J. Soil Sci. Plant Nutr. 24, 6760–6774. doi: 10.1007/s42729-024-02002-630311153

[B147] Yang J. Schrader S. Tebbe C. C. (2024a). Legacy effects of earthworms on soil microbial abundance, diversity, and community dynamics. Soil Biol. Biochem. 190, 109294. doi: 10.1016/j.soilbio.2023.10929442574925

[B148] Yildirim E. Ekinci M. Turan M. Ağar G. Dursun A. Kul R. . (2021). Humic + fulvic acid mitigated Cd adverse effects on plant growth, physiology and biochemical properties of garden cress. Sci. Rep. 11, 8040. doi: 10.1038/s41598-021-86991-9 33850186 PMC8044109

[B149] Zhang J. Fang H. Zhao Y. Zheng Y. Jiang J. Gu X. (2023). Responses of soil nutrients and rhizosphere microbial communities of a medicinal plant Pinellia ternata to vermicompost. 3 Biotech. 13, 353. doi: 10.1007/s13205-023-03780-z 37810193 PMC10555985

[B150] Zhang J. Liu Y.-X. Zhang N. Hu B. Jin T. Xu H. . (2019). NRT1.1B is associated with root microbiota composition and nitrogen use in field-grown rice. Nat. Biotechnol. 37, 676–684. doi: 10.1038/s41587-019-0104-4 31036930

[B151] Zhang Y. Yuan H. Peng S. Wang Z. Cai S. Chen Z. . (2024). Vermicomposting preferably alters fungal communities in wasted activated sludge and promotes the production of plant growth-promoting biostimulants in the vermicompost. Chem. Eng. J. 495, 153232. doi: 10.1016/j.cej.2024.15323242574925

[B152] Zhao Q. (2015). A Study on Sludge Treatment Using Factory-Based and Open-Field Earthworm Systems (Harbin: Nanjing Agricultural University). Master’s thesis, Nanjing Agricultural University.

[B153] Zheng L. (2025). Constructing rhizosphere synthetic microbial communities to promote efficient crop nutrient uptake and utilization. Synth. Biol. J. 6, 1058. doi: 10.12211/2096-8280.2025-075

[B154] Zhong X.-Z. Zeng Y. Wang S.-P. Sun Z.-Y. Tang Y.-Q. Kida K. (2020). Insight into the microbiology of nitrogen cycle in the dairy manure composting process revealed by combining high-throughput sequencing and quantitative PCR. Bioresour. Technol. 301, 122760. doi: 10.1016/j.biortech.2020.122760 31972401

